# Genetic Mapping and QTL Analysis of Growth-Related Traits in *Pinctada fucata* Using Restriction-Site Associated DNA Sequencing

**DOI:** 10.1371/journal.pone.0111707

**Published:** 2014-11-04

**Authors:** Yaoguo Li, Maoxian He

**Affiliations:** 1 CAS Key Laboratory of Tropical Marine Bio-resources and Ecology, Guangdong Provincial Key Laboratory of Applied Marine Biology, South China Sea Institute of Oceanology, Chinese Academy of Sciences, Guangzhou, China; 2 University of Chinese Academy of Sciences, Beijing, China; Temasek Life Sciences Laboratory, Singapore

## Abstract

The pearl oyster, *Pinctada fucata* (*P. fucata*), is one of the marine bivalves that is predominantly cultured for pearl production. To obtain more genetic information for breeding purposes, we constructed a high-density linkage map of *P. fucata* and identified quantitative trait loci (QTL) for growth-related traits. One F1 family, which included the two parents, 48 largest progeny and 50 smallest progeny, was sampled to construct a linkage map using restriction site-associated DNA sequencing (RAD-Seq). With low coverage data, 1956.53 million clean reads and 86,342 candidate RAD loci were generated. A total of 1373 segregating SNPs were used to construct a sex-average linkage map. This spanned 1091.81 centimorgans (cM), with 14 linkage groups and an average marker interval of 1.41 cM. The genetic linkage map coverage, Coa, was 97.24%. Thirty-nine QTL-peak loci, for seven growth-related traits, were identified using the single-marker analysis, nonparametric mapping Kruskal-Wallis (KW) test. Parameters included three for shell height, six for shell length, five for shell width, four for hinge length, 11 for total weight, eight for soft tissue weight and two for shell weight. The QTL peak loci for shell height, shell length and shell weight were all located in linkage group 6. The genotype frequencies of most QTL peak loci showed significant differences between the large subpopulation and the small subpopulation (*P*<0.05). These results highlight the effectiveness of RAD-Seq as a tool for generation of QTL-targeted and genome-wide marker data in the non-model animal, *P. fucata*, and its possible utility in marker-assisted selection (MAS).

## Introduction

The pearl oyster, *Pinctada fucata* (*P. fucata*), is a marine bivalve found in China and Japan, where it is predominantly cultured for pearl production. In China, the regions where it is cultured are mainly distributed along three coastal provinces (Guangdong, Guangxi and Hainan) of the South China Sea. Artificial breeding may no longer be commercially sustainable due to heavy mortality and the decline of pearl quality observed in established breeding programs [1]. Many factors, such as inbreeding [2], disease outbreak [3] and environmental threats [4], have led to the genetic degeneration and high mortality of *P. fucata*. Solutions to genetic degeneration include the use of conventional breeding methods, selection, crossbreeding and hybridization to improve the quality of aquaculture strains [5]. For *P. fucata*, breeding with the aim of high quality pearl production, for example the selection of pearl color, weight [6] or shell traits [1], has been conducted and gave positive results. Another solution to the degeneration of *P. fucata* is marker-assisted selection (MAS). Molecular genetic polymorphisms can be integrated with artificial selection of phenotypes by applying MAS to achieve substantial increases in the efficiency of artificial selection [7]. The basic methodology of MAS is high-density linkage map construction and quantitative trait loci (QTL) detection. Genetic linkage maps are constructed by assigning polymorphic DNA markers to chromosome configurations [8]. In shellfish, linkage maps of *Crassostrea gigas* [9], *Argopecten irradians* [10], *Pinctada maxima* [11] and *Pinctada martensii* [12] have been constructed. The availability of genetic linkage maps makes it possible to identify QTLs in aquaculture species and assists in the selection of desired traits, such as disease resistance, sex determination and growth traits [13]. Quantitative trait loci in many aquaculture species, such as *Crassostrea gigas* (disease resistance) [14], *Argopecten irradians* (growth) [10] and *Nodipecten subnodosus* (orange shell color) [15], have been identified. However, studies of single nucleotide polymorphisms (SNP) have rarely been reported [16], [17] and no QTLs have been identified in *P. fucata*. Therefore, it is important to identify new SNPs and QTLs that are associated with growth-related traits, which may contribute to the improvement of pearl production in *P. fucata*.

The application of restriction fragment length polymorphism, randomly amplified polymorphic DNA, amplified fragment length polymorphism, simple sequence repeats, SNPs and expressed sequence tags has changed the approach to genetic research in the field of aquaculture and has allowed the construction of genetic linkage maps [18]. Among these markers, SNPs are the most abundant type found in the genome and are suitable for automated large-scale genotyping [19]. There are over 30 different methods that are used for the genotyping of SNPs [20]. For next-generation sequencing methods, restriction-site associated DNA sequencing (RAD-Seq) creates a reduced representation of the genome. It combines the use of restriction enzymes and molecular identifiers with Illumina sequencing to facilitate SNP discovery and high-throughput genotyping of large populations [21], [22]. This technique was first applied to the genetic analysis of lateral plate armor in *Gasterosteus aculeatus*. Markers at the *Eda* locus, linked to plate loss, were identified in linkage group (LG) IV [22]. In aquaculture species, RAD-Seq was used to construct a linkage map for *Gnathopogon* [8] and to detect SNPs linked to QTLs for infectious pancreatic necrosis resistance in Atlantic salmon [21]. The RAD-Seq data can be readily analyzed without any prior genetic information, which makes the technique applicable to *P. fucata* for large-scale SNP discovery.

Methods that are frequently used for QTL mapping include the Kruskal–Wallis (KW) non-parametric test, interval mapping (IM) and composite interval mapping [10], [23], [24]. The KW nonparametric test is equal to the nonparametric version of a one-way analysis of variance [24], which enables the detection of individual association between markers and traits [25], [26]. An improved method, developed by Lander and Botstein [27], is IM. This method determines the likelihood for the presence of a segregating QTL at each position in the genome. The genetic effects of the QTL and the residual variance are also calculated when IM is used [25]. In contrast to single-marker association KW nonparametric analysis, IM allows one to detect QTL effects at every map position, rather than just the marker positions [28]. Based on IM, composite interval mapping was developed to eliminate the bias in QTL parameter estimation, reduce the residual error variance and increase the power of QTL detection [29]. The KW nonparametric test is normally used for QTL analysis when no assumptions are made about the probability distribution(s) of the quantitative trait [15], [30]. For bulk segregant studies (using individuals from the tails of the phenotypic distribution) based on high-throughput sequencing, a statistical framework for QTL mapping was described, and accelerated the identification of QTLs in both model and non-model organisms [31], [32].

The aim of this study was to identify a large number of SNP markers from the *P. fucata* genome using the RAD-seq method, with particular focus on high density genetic linkage map construction and growth-related QTL detection. In this analysis, we built a high-density sex-average linkage map of *P. fucata* using 1373 SNPs identified from RAD-seq. We also identified 39 QTL-peak loci for growth-related traits and found that the genotype frequencies were significantly different between the large and small subpopulations (*P*<0.05). These identified QTLs may be valuable resources for MAS in *P. fucata*.

## Materials and Methods

### Ethics Statement

All animal work has been conducted according to relevant national and international guidelines. No specific permissions are required to work with invertebrates in China. Similarly, no specific permissions were required for the collection of *P. fucata* from sample sites because they were not collected from protected areas of land. The pearl oyster *P. fucata*, is not an endangered or protected species.

### Mapping family and trait measurement

The breeding program of *P. fucata* was carried out at the Marine Biology Research Station, Daya Bay, Chinese Academy of Sciences, Shenzhen, China. A female and male adult *P. fucata* were obtained from a wild population in Dapeng Bay (22°41′N–22°61′N, 114°26′E–114°51′E), Shenzhen, China. These were used as founders of an F1 intercross. The hinge length (HL) of 1047 6-month-old F1 progeny of the family was measured. Seventy four of the largest (HL >33 mm) and 74 of the smallest oysters (HL <19.7 mm) were sampled. Each oyster was numbered and shell height (SH), shell length (SL), shell width (SW), HL, total weight (Wt), soft tissue weight (Wf), and shell weight (Ws) were measured. The adductor muscle or the whole soft tissue was also sampled and preserved in 90% ethanol for DNA extraction. A total of 100 individuals, which included the two parents, the 48 largest individuals (defined as the large subpopulation) and the 50 smallest individuals (defined as the small subpopulation) were then used for linkage and QTL mapping.

### RAD library construction and genotyping

Genomic DNA of the two parents and the 98 F1 progeny was extracted from the preserved samples using E.Z.N.A mollusc DNA Kit (Omega,USA), in accordance with the manufacturer’s instructions. The concentration of extracted DNA was estimated with a spectrophotometer, using OD_260/280_, and DNA quality was analyzed using agarose gel electrophoresis. The RAD library construction procedure closely followed the methodology described by Baird *et al* [22]. Briefly, genomic DNA was digested at 37°C for 30 min with the restriction endonuclease *Eco*RI (Takara, Dalian, China). The reactions were heat inactivated at 65°C for 20 min. Modified Illumina adapters (P1 adapters) that contained barcode sequences were added to the samples, along with 1 µL of 100 mM rATP (Promega), 1 µL 10× *Eco*RI buffer, 0.5 µL (1000 U) T4 DNA Ligase (New England Biolabs) and 5 µL H_2_O. The reactions were incubated at room temperature for 20 min and then heat inactivated at 65°C for 20 min. The ligation products were then combined in appropriate multiplex pools. For each library pool, digested DNA was sheared to an average length of 500 bp. Each of the 22 library samples were then separated by electrophoresis through a 1.3% agarose gel and fragments in the 300–700 bp range were isolated using a MinElute Gel Extraction kit (Qiagen). The Quick Blunting Kit (New England Biolabs) was used to polish the ends of the DNA. The samples were re-purified and 15 U of Exo-Klenow (Enzymatics) were used to generate adenine overhangs on the 3′ end of the DNA, at 37°C. After subsequent purification, 1 µL of 10 µM P2 adapters (a divergent modified Solexa adapter, Illumina) were ligated to the DNA products. Samples were re-purified and eluted in 50 µL. Five microliters of this product were used in PCR amplification with 50 µL of Phusion Master Mix (New England Biolabs), 5 µL of 10 µM modified Solexa Amplification primer mix (Illumina) and 40 µL of H_2_O. Phusion PCR settings followed product guidelines for a total of 16–18 cycles. Samples were gel purified, to excise the DNA fragments (300–700 bp), and dissolved in Elution Buffer. After quality and quantity tests on the PCR products, the obtained RAD libraries were sequenced on an Illumina Hiseq 2000 platform. For each parent, sequencing data size was set to obtain twice as much of the genome. For each progeny, sequencing data size was set to obtain as much as half of the genome. Raw Illumina reads were filtered to eliminate adapter sequences. The reads with a low quality (Q≤5 E) base number of greater than half of the total number of nucleotides and reads that did not show the enzyme cleaved sequence, “AATTC”, in the first five bases were eliminated. Sequences were sorted to individuals in accordance with the barcode sequences. They were then used for clustering analysis (a minimum stack depth of two) and genotyping by the software stacks [33]. The genotype of a locus was also determined by the maximum likelihood method.

### Linkage map construction and analysis

The SNP markers were categorized into “lm×ll”, “nn×np” and “hk×hk” types, which represent heterozygosity in the maternal, paternal and both parents, respectively. Markers that were genotyped in less than 85% of progeny and markers that showed significantly distorted segregation (χ^2^ test, *P*<0.05) were excluded. All of the marker information was input into JoinMap 4.0 [34] and the genetic analysis was carried out under the cross pollination population type. Similar markers were eliminated using the function “Similarity of Loci” and loci that segregated only in the paternal or maternal population were used for sex-specific linkage map construction, using the function “Create Maternal and Paternal Population Nodes”. The threshold independence logarithm of the odds (LOD) scores was set to 3.0–5.0 for the maternal map and 3.0–7.0 for the paternal map. The “lm×ll”, “nn×np” and “hk×hk” RAD-tag markers that were genotyped in more than 85% of progeny and segregated in a Mendelian manner were used to create the sex-average linkage map by JoinMap 4.0, with the threshold independence LOD score set to 4.0–6.0. Map distances (in cM) were calculated using Kosambi’s mapping function [35]. To calculate genome coverage of the linkage maps, observed genome length (Goa) and expected genome length (Ge) need to be established. The Goa was taken as the total length of all markers on the framework map and included the triplets and doublets [36]. Two approaches were used to estimate Ge of the linkage map: (1) Ge1 was calculated by adding 2 s (s was the average spacing of the genetic linkage map) to the length of each genetic linkage group, to account for chromosome ends [37]. (2) Ge2 was calculated by multiplying the length of each genetic linkage group by (m+1)/(m−1), where m was the number of loci in each genetic linkage group [38]. The average of Ge1 and Ge2 was used as Ge, to describe the genome length. Genome coverage (Coa) was denoted by Goa/Ge [39].

### QTL detection and genotype frequency comparison

Pearson correlation coefficients were used to determine the linear correlation between pairs of growth-related traits, using software SPSS version 16.0 (SPSS Inc., USA). The mean size of growth-related traits was compared in the large and small subpopulation using the Student’s t-test in SPSS version 16.0. The significance level was set to 0.05. The QTL mapping was performed using MapQTL 5 software [25] and the KW non-parametric test was performed to determine the significant relationship between the regions of the genome and growth-related traits. We used the nonparametric KW analysis because the growth-related traits of the individuals used in this study deviated from a normal distribution, and no assumptions were being made about the probability distributions of the quantitative traits. The KW test ranks all individuals in accordance with the quantitative trait and classifies them in accordance with their marker genotype. A KW value larger than the thresholds given by the KW test (χ^2^ test, *P*<0.005) and a degree of correlation between loci and traits that was equal to or greater than four asterisks were used to identify QTL-peak loci [25]. The genotype frequency difference between the large subpopulation and the small subpopulation was compared using the Chi-square test in SPSS version 16.0. The significance level was set to 0.05.

## Results

### RAD sequencing and genotyping

There are few known SNP markers available for the linkage mapping of *P. fucata*. Hence, the RAD-seq analysis was employed for DNA polymorphism detection and genotyping. The Illumina Hiseq 2000 sequencing yielded a total of 84.36 gigabases (Gb) of clean bases from the 100 samples. The average number of clean bases per individual was 0.84 Gb. Sample 9 was the individual with least number of clean bases (0.45 Gb) and sample 2 was the individual with maximum number of clean bases (1.93 Gb) (**Table S1**). A total of 1956.53 million clean reads was generated and 86,342 candidate RAD loci were identified. After 84,336 candidate RAD loci were discarded (were scored in less than 85% of the progeny or were types of marker that had “ab×cd” for four alleles and “ef×eg” for three alleles), 2006 “lm×ll”, “nn×np” and “hk×hk” type of SNP markers, which were scored in enough individuals, were retained for further analysis. Amongst the 2006 markers, 625 showed deviation from Mendelian segregation (*P*<0.05). A total of 1381 SNP markers, which included 562 “lm×ll” type markers, 611 “nn×np” type markers and 208 “hk×hk” type markers, were retained for linkage map construction.

### Linkage analysis

A total of 562 “lm×ll” type SNP markers were used for the female linkage map construction. After four similarity markers were discarded, 558 markers were located on the female linkage map. Fourteen linkage groups were identified and the number of loci per linkage group varied from nine to 94, with a mean of 39.8. The female linkage map spanned 1024.31 cM, with an average spacing of 3.19 cM and a Coa of 93.51% (**Table 1** **, Figure S1**).

**Table 1:** Summary of the female linkage map for *Pinctada fucata.*

Linkage group	Number of markers	Length (cM)	Average marker interval (cM)
1	79	128.25	1.64
2	88	68.80	0.79
3	27	60.88	2.34
4	17	89.10	5.57
5	94	84.80	0.91
6	76	51.08	0.68
7	47	80.77	1.76
8	14	39.58	3.04
9	15	73.30	5.24
10	22	81.32	3.87
11	42	79.00	1.93
12	16	64.94	4.33
13	9	44.18	5.52
14	12	78.31	7.12
Average	39.8	73.16	3.19
Total	558	1024.31	

For the construction of the male linkage map, 611 “nn×np” type SNP markers were used and four similarity markers were excluded. The remaining 607 markers were all located on the linkage map. Seventeen linkage groups were identified, which included two triplets and one doublet. The number of loci per linkage group varied from two to 158, with a mean of 35.7. The male linkage map covered 928.00 cM, with an average spacing of 4.03 cM and a Coa of 90.67% (**Table 2** **, Figure S2**).

**Table 2:** Summary of the male linkage map for *Pinctada fucata.*

Linkage group	Number of markers	Length (cM)	Average marker interval (cM)
1	158	119.61	0.76
2	76	45.04	0.60
3	23	86.41	3.93
4	16	26.96	1.80
5	66	105.59	1.62
6	63	122.30	1.97
7	61	48.55	0.80
8	39	79.10	2.08
9	5	9.14	2.29
10	24	54.83	2.38
11	3	35.65	17.83
12	12	60.97	5.54
13	22	63.63	3.03
14	3	25.94	12.97
15	21	18.32	0.92
16	13	17.25	1.44
17	2	8.71	8.71
Average	35.7	54.58	4.03
Total	607	928.00	

**Figure 1:**
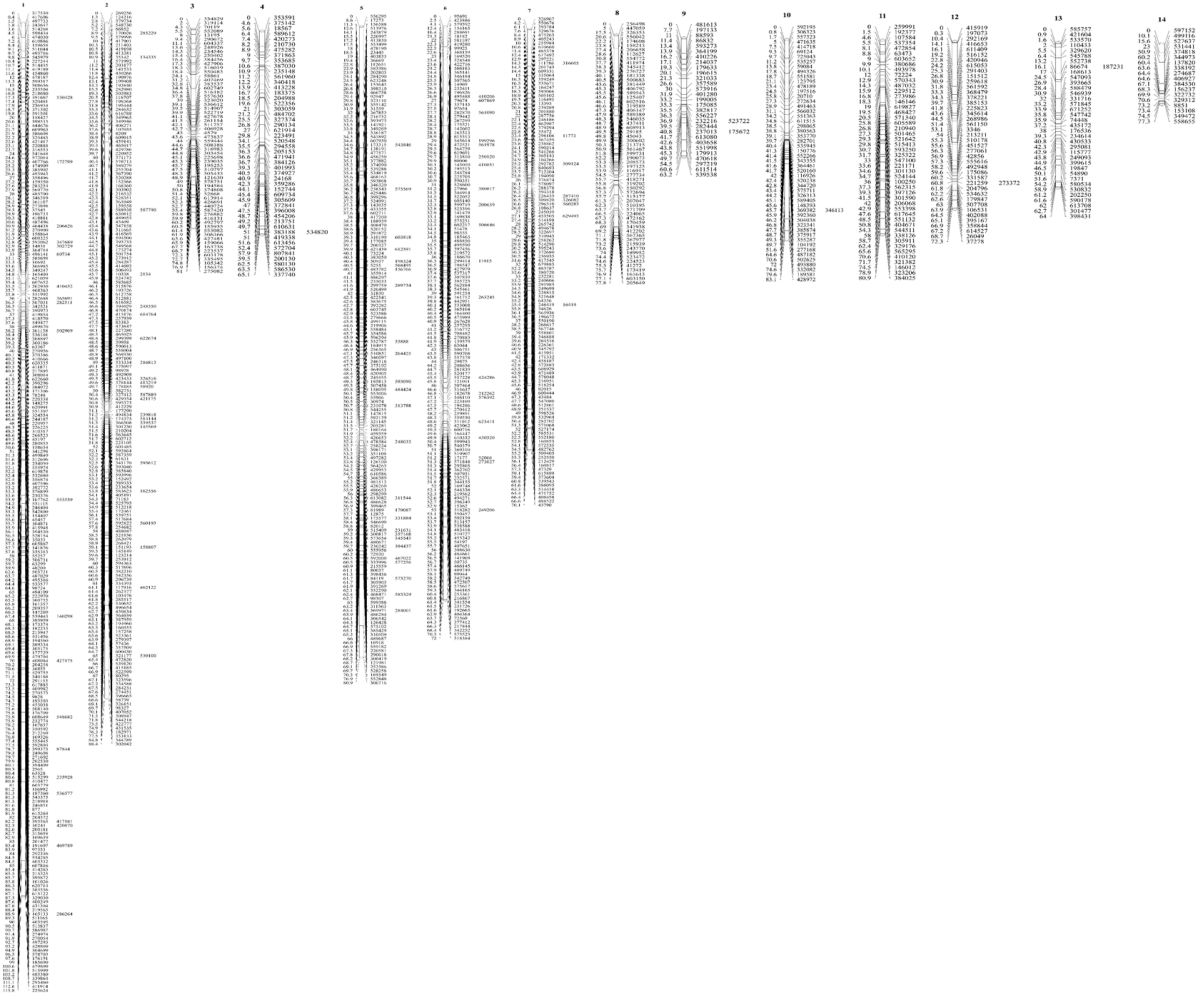
The sex-average linkage map for *P. fucata*. The map is composed of 14 linkage groups, with 1373 SNP markers, and spans 1091.81 cM. The map distances (in cM) are indicated on the left of the chromosomes and the names of the SNP markers are shown on the right.

Five hundred and fifty-eight “lm×ll” type SNP markers, 607 “nn×np” type SNP markers and 208 “hk×hk” type SNP markers were used for construction of the sex-average map. All of the 1373 markers (**Table S2**) were located on the linkage map. Fourteen linkage groups were formed and the number of markers per linkage group varied from 18 to 265, with a mean of 98. The sex-average linkage map covered 1091.81 cM, with an average spacing of 1.41 cM. The length of the linkage groups ranged from 63.22 cM to 115.79 cM, with an average of 77.98 cM. (**Table 3** **,** **Figure 1**). The expected genome length, Ge, was 1122.7 cM for the sex-average linkage map, with an expected genome length, Ge1, of 1114.3 cM and Ge2 of 1131.1 cM. The genetic linkage map coverage, Coa, was 97.24%. Given the estimated genome size of 1150 Mb for *P. fucata* [40], the average recombination rate across all of the linkage groups was 0.97 cM/Mb.

**Table 3:** Summary of the sex-average map for *Pinctada fucata.*

Linkage group	Number of markers	Length (cM)	Average marker interval (cM)
1	265	115.79	0.44
2	206	88.37	0.43
3	63	81.00	1.31
4	50	65.05	1.33
5	193	80.88	0.42
6	176	72.04	0.41
7	130	70.05	0.54
8	61	77.82	1.30
9	31	63.22	2.11
10	53	83.10	1.60
11	48	80.87	1.72
12	42	72.30	1.76
13	37	64.00	1.78
14	18	77.32	4.55
Average	98	77.98	1.41
Total	1373	1091.81	

The sex-average linkage map contained all the markers located in the female and male linkage maps. Some LGs in the sex-average map contained mainly SNP markers from the corresponding female and male LGs. For example, LG 3 of the sex-average linkage map contained 64 SNP markers, which included 27 markers from LG 3 (28 markers) of the female map and 23 markers of LG 3 (24 markers) of the male map. The SNP markers in LG 3 of the sex-average linkage map also presented a similar order of markers as in the female and male linkage maps.

### Phenotypic values and correlation between growth-related traits

Pearson correlation coefficients between the growth-related traits ranged from 0.935 to 0.994 (**Table 4**), showing the growth-related trait was significantly correlated each other (*P*<0.01), and the mean value of each growth-related trait in the large subpopulation was significantly larger than that in the small subpopulation (*P*<0.0001) (**Table 5**).

**Table 4:** Pearson correlation coefficients between growth-related traits measured on F1 progeny.

	SH	SL	SW	HL	Wt	Wf	Ws
SH	1	0.988	0.970	0.972	0.970	0.961	0.972
SL		1	0.975	0.984	0.972	0.954	0.976
SW			1	0.970	0.973	0.958	0.971
HL				1	0.958	0.935	0.965
Wt					1	0.975	0.994
Wf						1	0.967
Ws							1

All correlations are statistically significant at the 1% level. SH: Shell height, SL: shell length, SW: shell width, HL: hinge length, Wt: total weight, Wf: soft tissue weight, Ws: shell weight.

**Table 5:** Statistics of growth-related traits in the large and small subpopulations.

Growth traits	Large subpopulation	Small subpopulation	*P*-value
Shell height (mm)	33.95±3.33	17.02±1.66	0.0000
Shell length (mm)	37.55±3.13	20.51±1.48	0.0000
Shell width (mm)	11.22±1.26	5.80±0.64	0.0000
Hinge length (mm)	34.23±2.96	18.36±1.14	0.0000
Total weight (g)	4.39±1.12	0.69±0.17	0.0000
Soft tissue weight (g)	1.36±0.47	0.15±0.06	0.0000
Shell weight (g)	2.15±0.52	0.37±0.09	0.0000

Estimates are given as mean±standard deviation. *P*-values were statistically significant at the 0.05 level.

### QTL detection

To identify QTL-peak loci for growth-related traits, the phenotypic and genotypic data from the selected individuals were analyzed using the MapQTL5 software [25]. The statistical analysis was conducted by a nonparametric genomic scan based on the KW non-parametric test, where markers were assessed for an association with trait data. Thresholds given by the KW statistic for the QTL detection of SH, SL, SW, HL, Wt, Wf and Ws (*P*<0.005) were 10.913, 12.579, 11.082, 10.885, 7.898, 8.115 and 11.303, respectively. A total of 39 QTL-peak loci for growth-related traits were identified, which included three for SH, six for SL, five for SW, four for HL, 11 for Wt, eight for Wf and two for Ws (**Table 6**). All of the QTL-peak loci for SH, SL and Ws were located on LG 6 and Pearson correlation coefficients between pairs of the three growth-related traits were higher than between most of the other trait pairs (**Table 4**). The QTL detected for other traits were located on LG 1, LG 5, LG 6, LG 7 and LG 10. Three loci were found to be consensus QTLs for five growth-related traits, which included locus 423886 (SH, SL, SW, HL and Wt), locus 288691 (SH, SL, SW, HL and Wf) and locus 99925 (SL, SW, HL, Wt and Ws) (**Table 6**).

**Table 6:** QTL-peak loci for growth-related traits in the sex-average map.

Trait	Linkage group	QTL-peak locus	KW value	Map distance
Shell height	LG 6	423886	13.01	2.546
	LG 6	288691	12.487	16.446
	LG 6	337145	10.913	27.258
Shell length	LG 6	423886	17.862	2.546
	LG 6	288691	16.569	16.446
	LG 6	99925	16.511	22.484
	LG 6	410206	14.505	26.883
	LG 6	337145	13.689	27.258
	LG 6	546110	13.292	47.135
Shell width	LG 5	425446	11.082	36.681
	LG 5	291872	11.480	38.938
	LG 6	423886	12.587	2.546
	LG 6	288691	13.161	16.446
	LG 6	99925	11.943	22.484
Hinge length	LG 6	423886	13.307	2.546
	LG 6	288691	12.205	16.446
	LG 6	99925	11.312	22.484
	LG 7	373604	11.906	59.371
Total weight	LG 5	466758	11.068	28.641
	LG 5	291872	8.455	38.938
	LG 5	428260	8.378	55.478
	LG 5	298299	8.474	55.863
	LG 6	423886	9.678	2.546
	LG 6	154165	10.294	12.419
	LG 6	99925	9.580	22.484
	LG 6	410206	9.747	26.883
	LG 6	337145	9.295	27.258
	LG 10	306323	9.159	0.773
	LG 10	471635	7.898	5.030
Soft tissue weight	LG 1	196733	8.115	29.923
	LG 1	411871	8.281	40.465
	LG 5	291872	8.279	38.938
	LG 5	428260	8.229	55.478
	LG 6	288691	10.068	16.446
	LG 6	410206	9.076	26.883
	LG 6	337145	8.781	27.258
	LG 7	471489	12.269	43.867
Shell weight	LG 6	154165	13.770	12.419
	LG 6	99925	12.133	22.484

### Genotype frequency comparison

The genotype frequencies of all QTL-peak loci (except locus 196733 and locus 411871) showed significant differences (*P*<0.05) between the large subpopulation and the small subpopulation (**Table 7**). For example, the genotype frequencies for QTL-peak locus 423886 in the large subpopulation were 58% for the “np” type and 25% for the “nn” type. In the small subpopulation, the frequency for the “np” type was 26%, whilst the frequency for the “nn” type was 60%. The genotype frequencies of QTL-peak locus 466758 in the large subpopulation were 58% for the “hk” type, 12% for the “hh” type and 21% for the “kk” type. In the small subpopulation, the genotype frequencies were 32% for the “hk” type, 38% for the “hh” type and 16% for the “kk” type.

**Table 7:** Comparison of QTL-peak loci genotype frequency between the large and small subpopulations.

QTL-peak locus	Genotype frequency		*P*-value
	Large subpopulation	Small subpopulation	
423886	np (0.58) nn (0.25)	np (0.26) nn (0.60)	0.000
288691	np (0.27) nn (0.58)	np (0.58) nn (0.28)	0.001
337145	np (0.23) nn (0.60)	np (0.46) nn (0.42)	0.021
99925	np (0.54) nn (0.33)	np (0.28) nn (0.58)	0.007
410206	np (0.21) nn (0.56)	np (0.50) nn (0.42)	0.012
546110	np (0.31) nn (0.56)	np (0.54) nn (0.34)	0.017
425446	lm (0.31) ll (0.56)	lm (0.52) ll (0.32)	0.016
291872	lm (0.52) ll (0.35)	lm (0.26) ll (0.58)	0.009
373604	lm (0.52) ll (0.29)	lm (0.36) ll (0.58)	0.017
466758	hk (0.58) hh (0.12) kk (0.21)	hk (0.32) hh (0.38) kk (0.16)	0.006
428260	np (0.23) nn (0.65)	np (0.52) nn (0.34)	0.001
298299	lm (0.58) ll (0.31)	lm (0.32) ll (0.48)	0.022
154165	np (0.56) nn (0.27)	np (0.32) nn (0.54)	0.006
306323	lm (0.54) ll (0.35)	lm (0.28) ll (0.58)	0.009
471635	lm (0.31) ll (0.54)	lm (0.58) ll (0.30)	0.007
196733	np (0.31) nn (0.56)	np (0.44) nn (0.40)	0.124
411871	np (0.54) nn (0.29)	np (0.42) nn (0.44)	0.138
471489	hk (0.31) hh (0.33) kk (0.17)	hk (0.36) hh (0.14) kk (0.40)	0.014

The genotype frequency difference is statistically significant at the 5% level. “ll”, homozygous genotype as in paternal; “lm”, heterozygous genotype as in maternal; “nn”, homozygous genotype as in maternal; “np”, heterozygous genotype as in paternal; “hh”, “kk”, homozygous genotype in F1 progeny, and “hk” types represent heterozygous genotype as in paternal or maternal.

## Discussion

### Linkage map construction and analysis

The sex-average linkage map has fourteen linkage groups, which is consistent with the number of haploid chromosomes of *P. fucata* [41]. For the sex-specific maps, fourteen linkage groups were identified in the female map, whilst seventeen linkage groups were constructed in the male map. This discrepancy between the number of linkage groups and the haploid number was also found in *Argopecten irradians* [10] and zebrafish [42]. The increase in number of linkage groups over the haploid number indicates that additional markers in several areas of the genome or a larger mapping population are required to reduce the linkage groups to the haploid chromosome number [43]. Linkage maps for many bivalve species, such as *Chlamys farreri* [44] and *Ostrea edulis* L [45], have been constructed. Although they provide valuable information for the breeding of bivalve species, their use for QTL identification may be limited by their low resolution (5 to 20 cM). The sex-average linkage map for *P. fucata* consists of 1373 markers, with an average spacing of 1.4 cM. It is denser than the linkage maps for *Pinctada martensii* and *Pinctada maxima* [11], [12]. Linkage maps have been constructed for *Pinctada martensii* using amplified fragment length polymorphism and microsatellite markers from an F1 family of two parents and 78 progeny [12]. The female map has an average interval of 14.9 cM and the male map has an average interval of 16.1 cM. The sex-average linkage map for *Pinctada maxima* was constructed from 887 SNPs, with an average spacing of 2.0 cM, using 335 individuals that belonged to eight families. Genotyping was performed using Illumina 3 k iSelect custom arrays [11]. The sex-average linkage map coverage, Coa, observed here was 97.24%, which is similar to the linkage map of *Chlamys farreri* (99.5%, constructed by a 2b-restriction site-associated DNA method, using two parents and 96 progeny) [46]. Such a dense linkage map contains detailed information on the genome of *P. fucata* and can be used as the basis for a better understanding of genetic structure in shellfish species.

The estimated genome length of the integrated linkage map for *P. fucata* was 1122.7 cM. This is shorter than the expected genome length of a consensus map for *Pinctada martensii* (1415.9 cM for the female map, with 110 markers, and 1323.2 cM for the male map, with 98 markers) [12]. Maps of low resolution are commonly longer than maps of high density, with low marker density being the most likely cause of the overestimation of genome size [47], [48]. In comparisons of high resolution genetic linkage maps, those with more markers may result in a longer genome length. The consensus map of *Chlamys farreri* (1551.9 cM, with 3806 markers) [46] is longer than linkage maps of *P. fucata*, in this study, and *Pinctada maxima* [11]. The RAD-seq method was available for high-resolution linkage map construction of *P. fucata* and this research has provided foundation materials for the utility of RAD-seq in non-model animals.

### Segregation distortion

In this study, 2006 SNPs were scored in enough individuals and belongs to “lm×ll”, “nn×np” and “hk×hk” type of SNP markers. Amongst the 2006 markers, 625 showed deviation from Mendelian segregation. One of the problems for linkage mapping is marker-segregation distortion, which has also been observed in *Ostrea edulis* and *Crassostrea gigas* [49], [50]. The deviation from Mendelian expectations could be the result of duplicated genes, deleterious alleles, unusual meiotic segregation distortions and transposable elements [51]–[54]. The cause of SNP loci segregation distortion in *P. fucata* may be the same as that observed in *Crassostrea gigas*, in which Mendelian segregation occurs during early developmental stages but is distorted during later development. These distortions are largely attributable to selection against recessive deleterious mutations of fitness genes that are closely linked to the markers [50]. However, markers with moderate segregation distortion have little effect on marker order or the length of the linkage map [55], [56]. To be conservative, only the markers that segregated in a Mendelian manner (*P<*0.05) were used for the linkage map construction of *P. fucata*.

### QTL analysis

The IM method from the MapQTL5 software [25] was initially used to identify QTLs for *P. fucata*. The use of this method will provide high significance thresholds when the phenotype distribution is skewed [27], [57]. In this study, a *P. fucata* family with 1047 progeny were produced, and individuals at the extremes of the phenotype distribution (largest individuals with the HL >33 mm and smallest individuals with the HL <19.7 mm) were selected for QTL mapping. The growth-related traits of the selected individuals deviated from a normal distribution, which meant that the permutation test of the IM method was not justified and the LOD value of significance thresholds was too large. The use of the IM method in this analysis failed to identify any regions that reached genome-wide significance. The KW nonparametric test for QTL detection does not assume a normal distribution of quantitative traits [24], [30]. It has been used for QTL analysis in many species, such as the investigation of total biomass and shell width of Pacific lion-paw scallop (*Nodipecten subnodosus*) [15], plant persistency of red clover [58], cane splitting of red raspberry (*Rubus idaeus*) [59] and low-temperature tolerance of wheat [60]. It ranks all individuals by the quantitative trait value and classifies them by their genotype. A segregating QTL (with a large effect) that is closely linked to the tested marker will result in large differences in average rank of the marker genotype classes [25]. To take full advantage of the RAD-seq data, a statistical framework for QTL mapping [31], [32] may be adopted for QTL identify in *Pinctada fucata* when a reference genome is available.

Although QTL analyses have been carried out for economically important traits in over 20 aquaculture species, most QTLs were only mapped in large spaces between markers [13]. Using randomly selected individuals or all individuals of a family as mapping population, the identified QTLs are usually evenly distributed across the linkage groups [23], [61]. To identify QTLs, the family structure is extremely important in the experimental design. For oysters with large full-sibling families, generally only individuals with extreme phenotypes are genotyped [62]. The selection of individuals with extreme traits focuses the analysis on the most genetically informative individuals, reduces the overall cost of genotyping and increases the experimental power [27], [62], [63]. Here, we used the extreme phenotype trait individuals for QTL mapping to see whether the identified QTLs will be concentratedly distributed across the LGs. Growth-related traits of *P. fucata* are of particular interest to researchers due to their correlations with pearl production. In this study, 39 QTL-peak loci for growth-related traits were identified and most of the QTL-peak loci were distributed on LG 6. Many QTL-peak loci, such as locus 423886, locus 288691 and locus 99925, were also found to be consensus QTLs for growth-related traits. The consensus QTLs may correspond to distinct, closely linked QTLs or one QTL that acts upon several quantitative characters involved in the same metabolic pathway [64]. Consensus QTLs have previously been observed in soybean, in which several QTLs for agronomic traits were mapped to the same loci [65]. The detection of concentratedly distributed and consensus QTLs in *P. fucata* highlight their further application for MAS.

### Genotype frequency comparison

Marker-trait association analysis is an alternative approach to QTL mapping that we used to identify QTLs for growth [66]. In this methodology, association is inferred by the comparison of allelic frequency differences between selected populations [67]. The large subpopulation and small subpopulation selected from the mapping family were used for allele frequency comparison. All growth-related traits had significantly higher mean values in the large subpopulation than in the small subpopulation (*P*<0.0001) and the genotype frequencies of most QTL-peak loci showed significant differences (*P*<0.05) between the two subpopulations. For example, the genotype frequencies at locus 423886 and locus 466758 showed significant differences between the two subpopulations. The frequency of the “np” genotype of locus 423886, plus “hk” and “kk” of locus 466758, were higher in the large subpopulation than in the small subpopulation. That may be caused by the accumulation of genotype frequency at marker-trait association loci during the selection of extreme individuals. These identified marker-trait association loci may be valuable resources for molecular selection breeding in *P. fucata*.

## Conclusions

The identification of high growth-related traits that may contribute to the improvement of pearl production is the main goal of genetic breeding programs of *P. fucata*. The RAD-seq method was successfully utilized for the identification of a large number of SNPs and the construction of a high-density genetic linkage map of *P. fucata*. The choice of extreme phenotype individuals from a large full-sib *P. fucata* family as the mapping population was able to identify QTLs by the KW non-parametric test. Many QTL-peak loci were found to be consensus loci for growth-related traits and were located at similar regions of the linkage groups. Significant differences in genotype frequency, for most of the QTL-peak loci, were also observed between the large and the small subpopulations. The choice of extreme phenotype individuals from a large full-sib *P. fucata* family as the mapping population could yield QTLs that concentrate distribution across the linkage groups. The location of QTLs at similar regions of the linkage groups and QTLs that act upon several quantitative characters highlight their further application for MAS in *P. fucata*.

## Supporting Information

Figure S1 **The female linkage map for** ***Pinctada fucata*** **.** The map is composed of 14 linkage groups, with 558 markers, and spans 1024.3 cM. The map distances (in cM) are indicated on the left of the chromosomes and the names of the SNP markers are shown on the right.
(DOC)

Figure S2 **The male linkage map for** ***Pinctada fucata*** **.** The map is composed of 17 linkage groups, with 607 markers, and spans 928.0 cM. The map distances (in cM) are indicated on the left of the chromosomes and the names of the SNP markers are shown on the right.
(DOC)

Table S1 **Statistics of sample information.** Detailed statistics on 100 samples used as the mapping family. Q20-rate means the percentage of bases with a quality value ≥20 and Q30-rate means the percentage of bases with quality value ≥30.
(DOC)

Table S2 **Markers used for linkage and QTL mapping.** The information of 1373 SNP markers includes marker name, SNP position, major allele, minor allele and the consensus sequences those contained the SNPs.
(XLS)
