# Supplementary figures and images for: Genetic Mapping and QTL Analysis of Growth-Related Traits in Pinctada fucata Using Restriction-Site Associated DNA Sequencing
Source: PLoS One. 2014 Nov 4;9(11):e111707. doi: 10.1371/journal.pone.0111707 (PMC4219768; doi:10.1371/journal.pone.0111707)

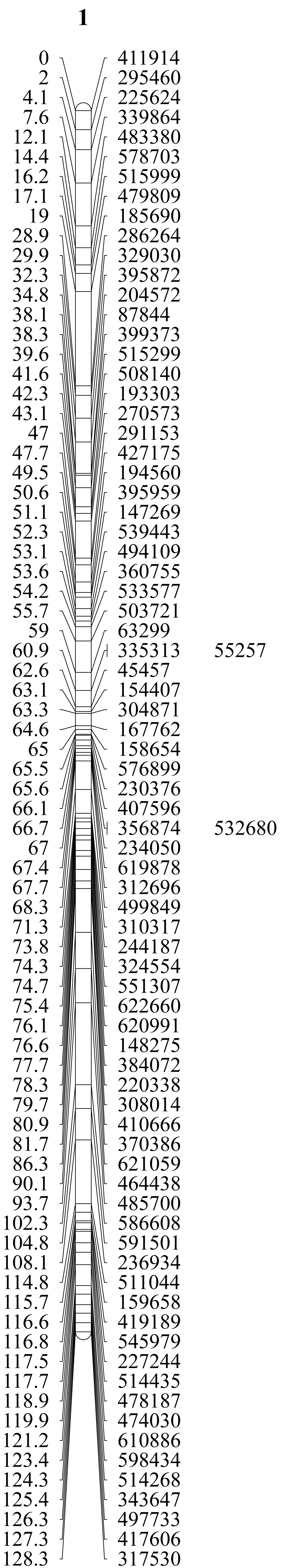

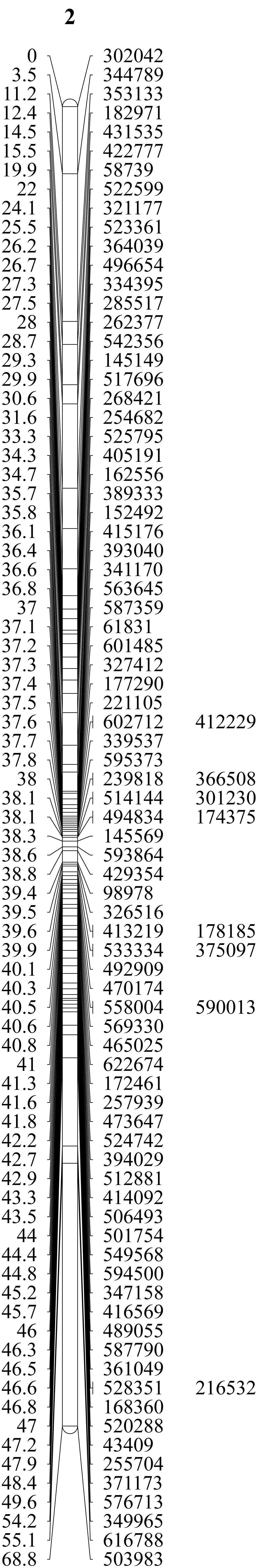

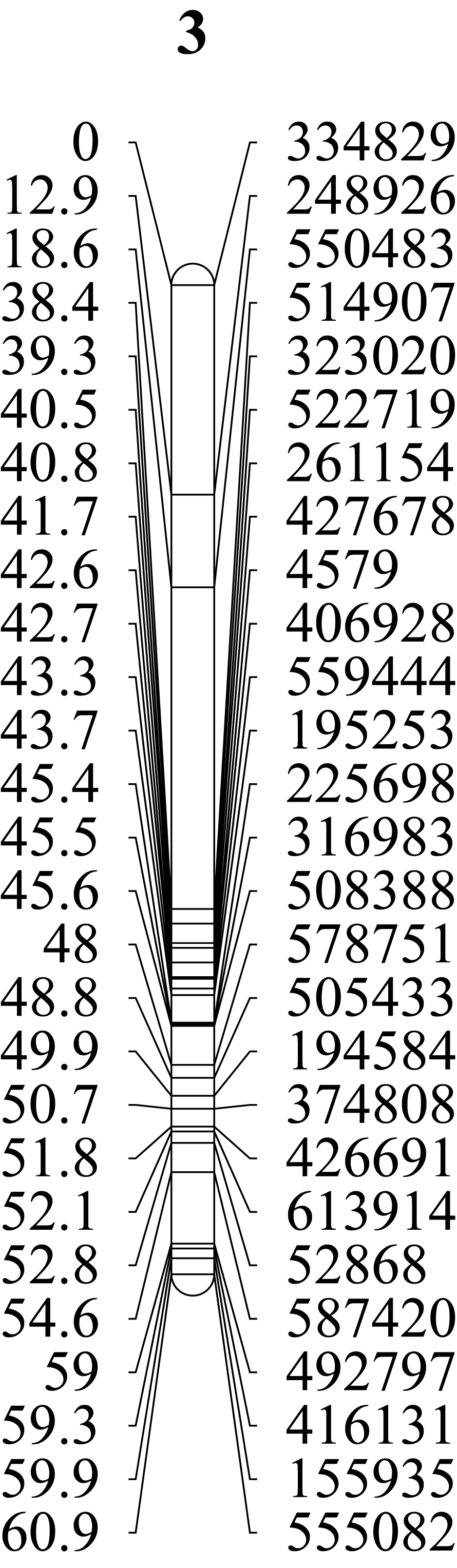

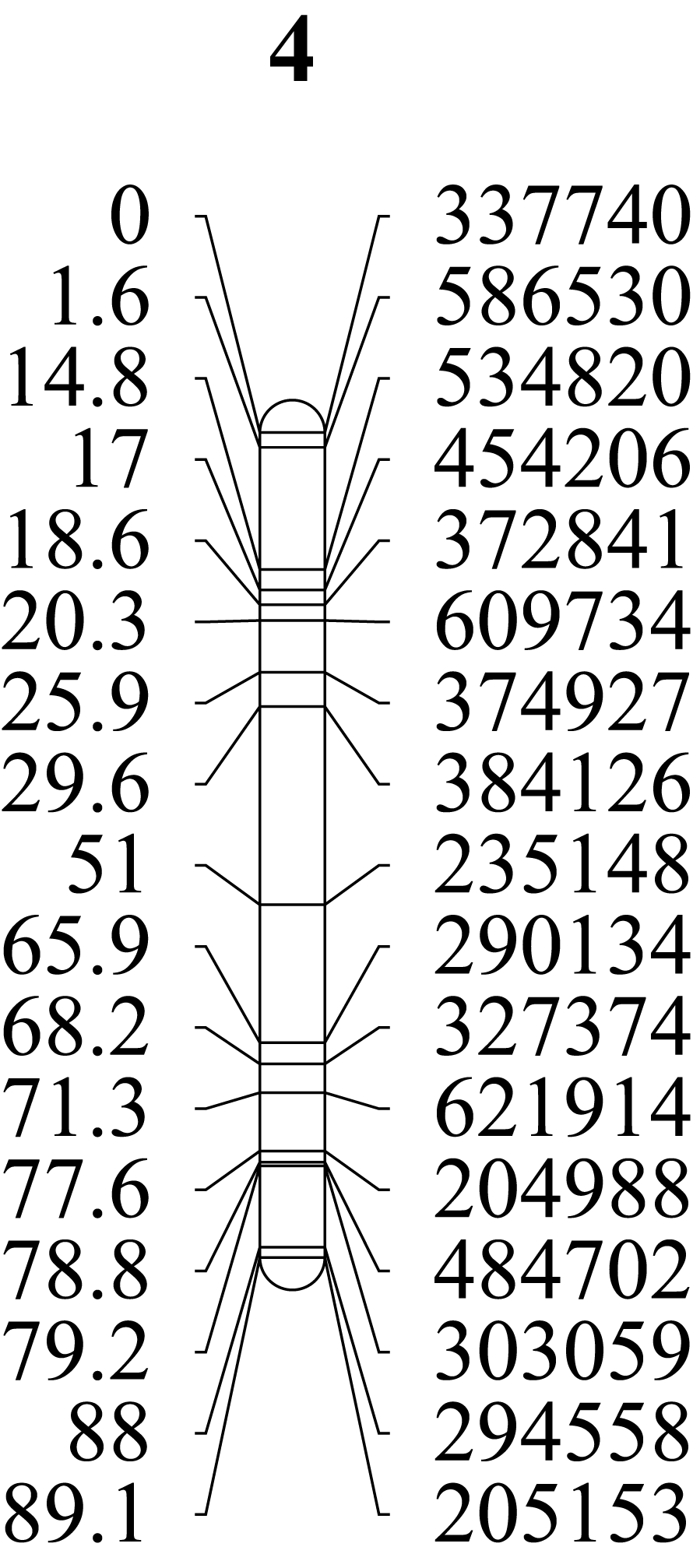


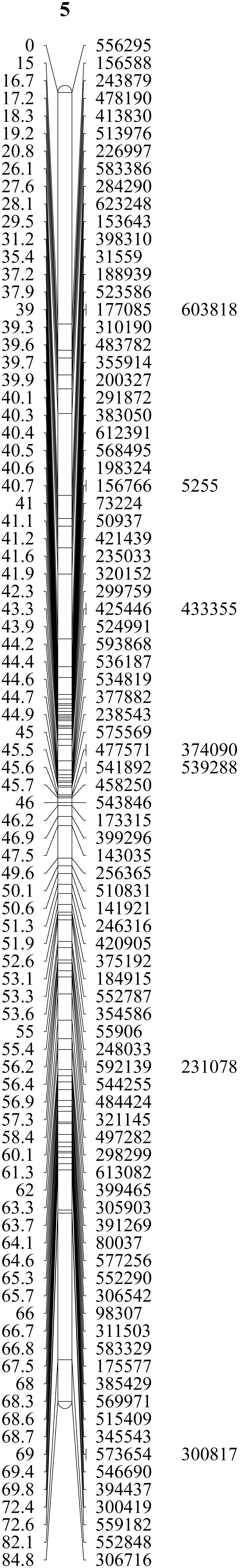

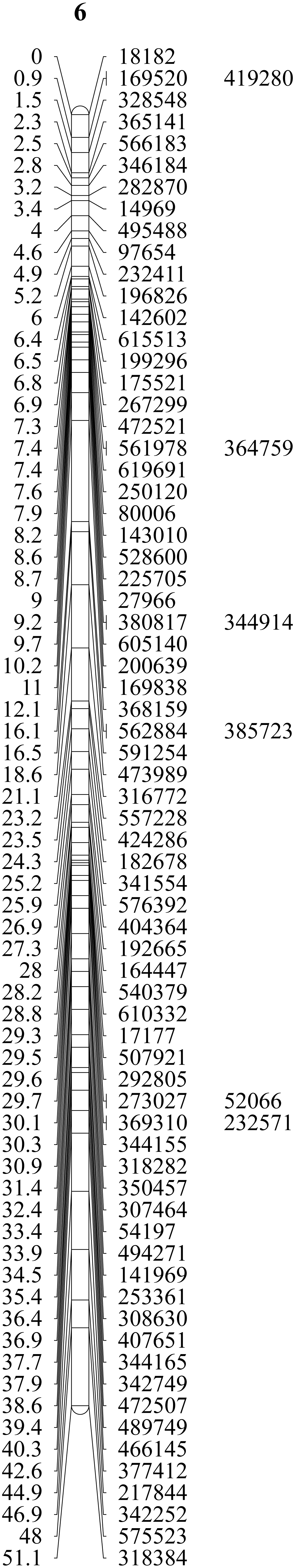

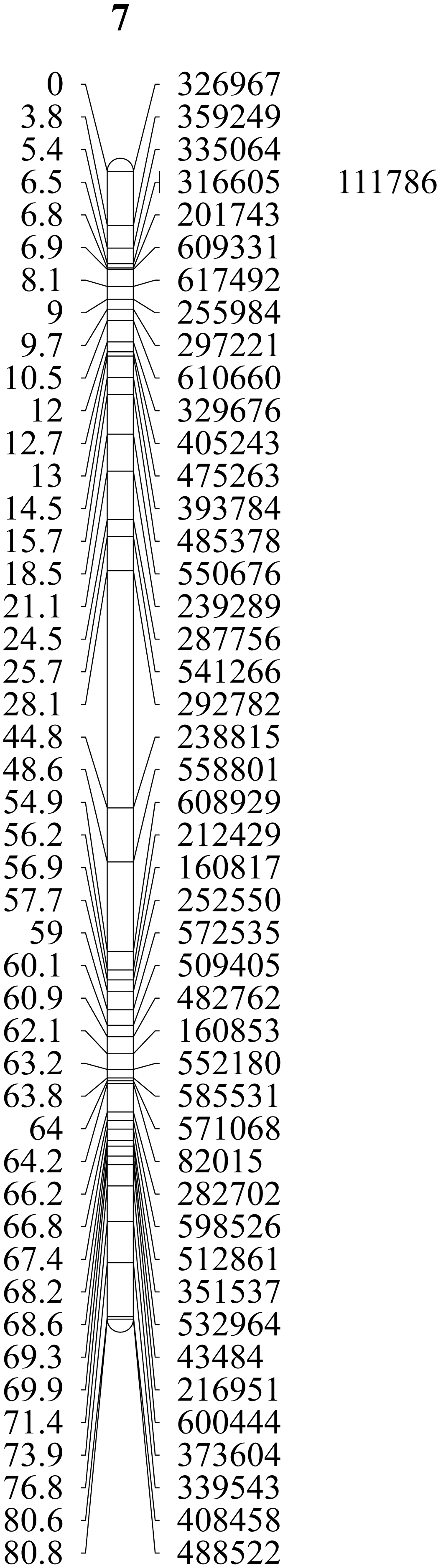

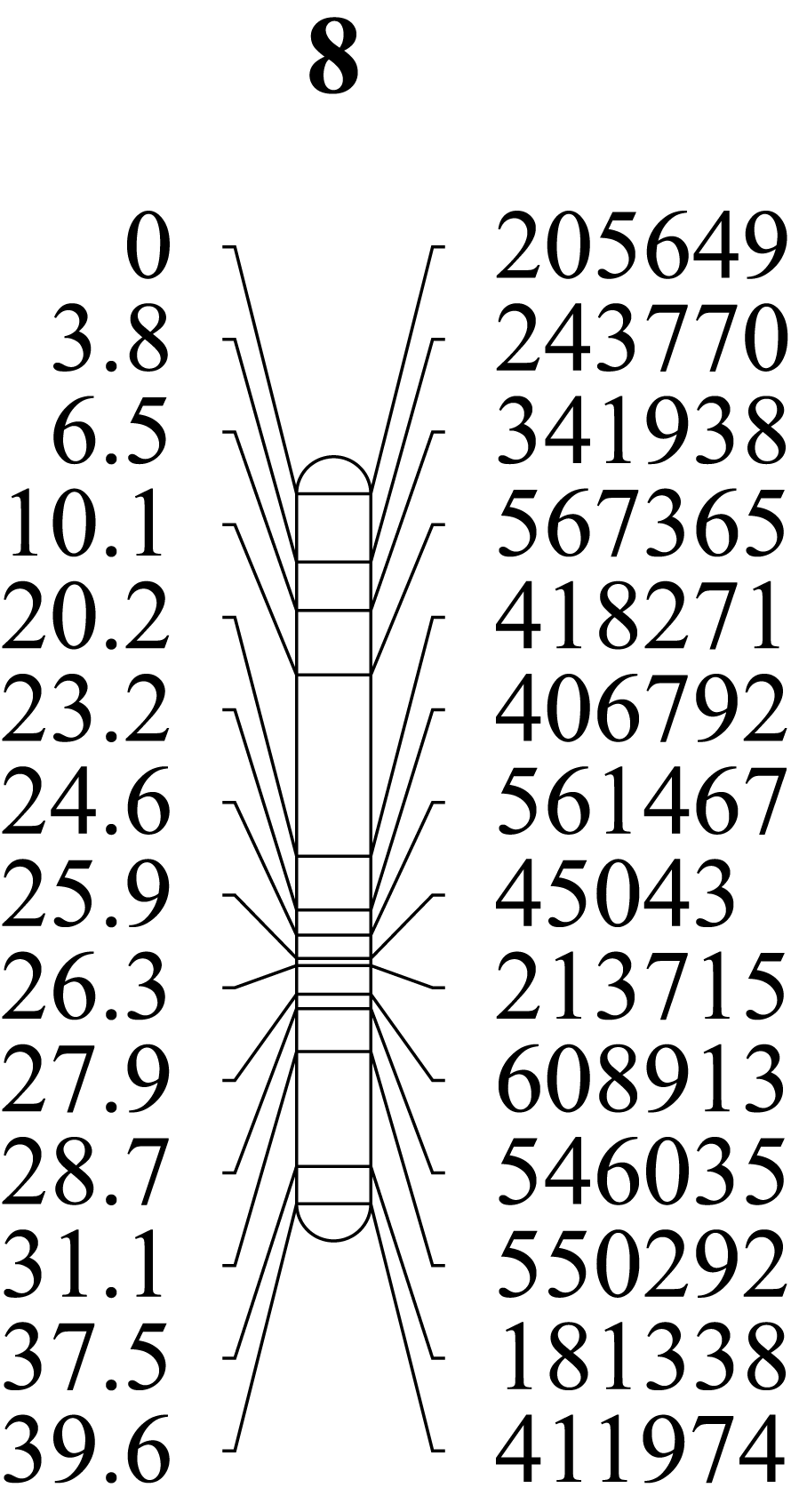


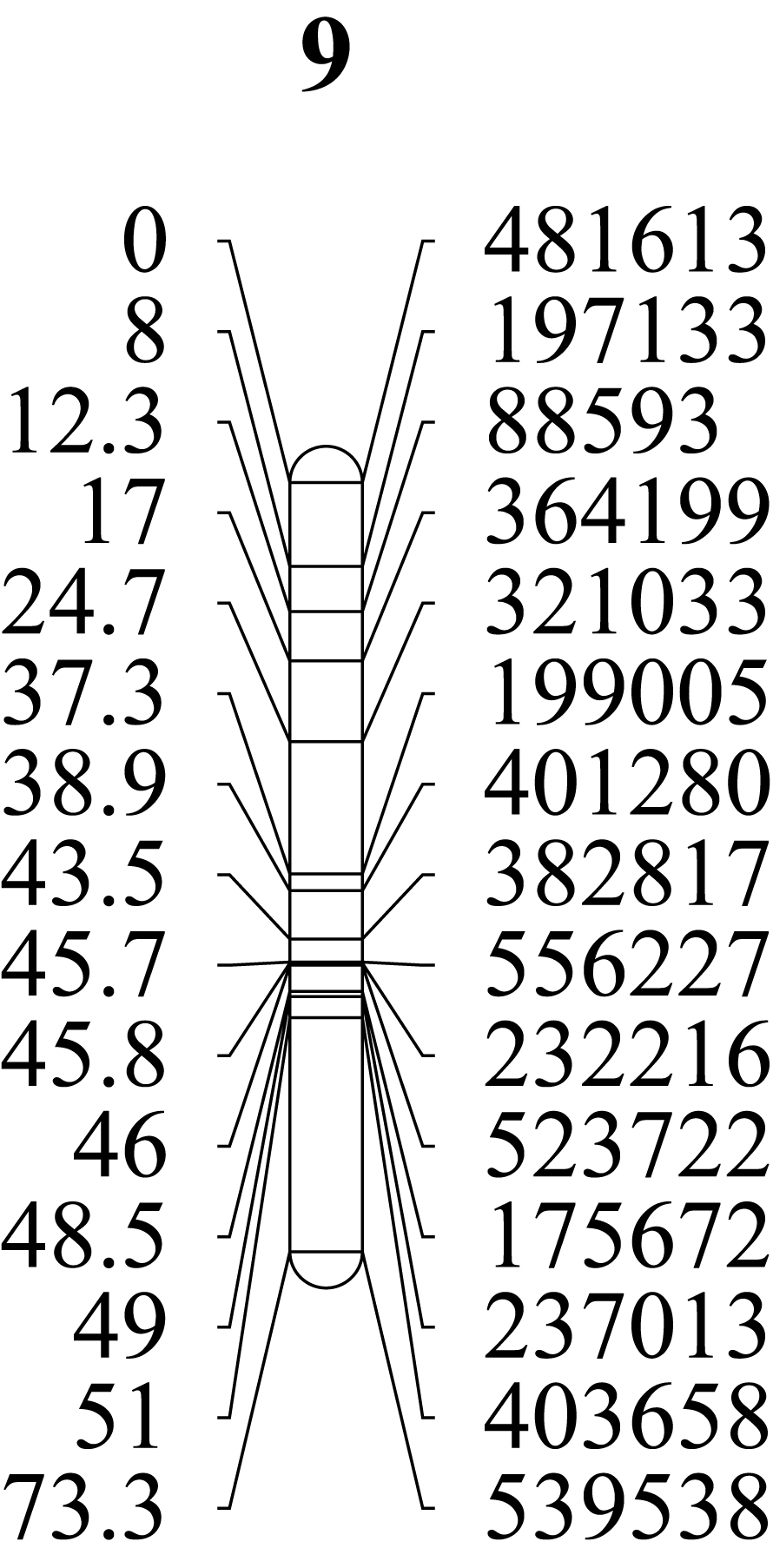

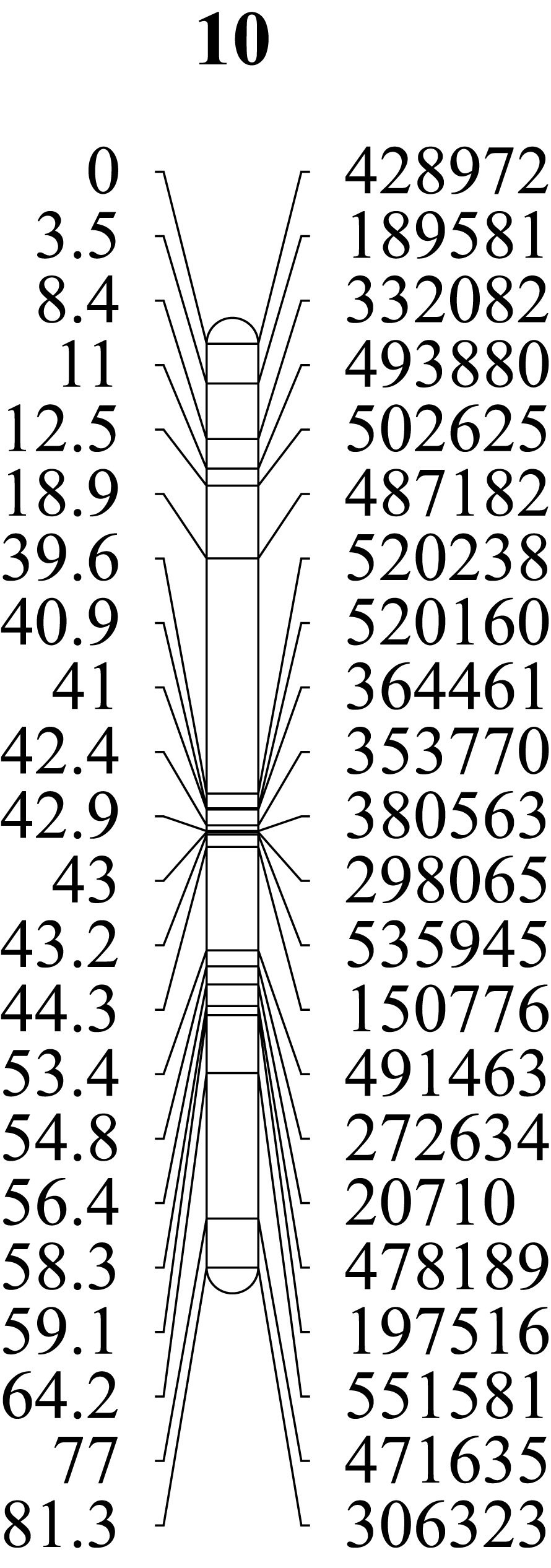

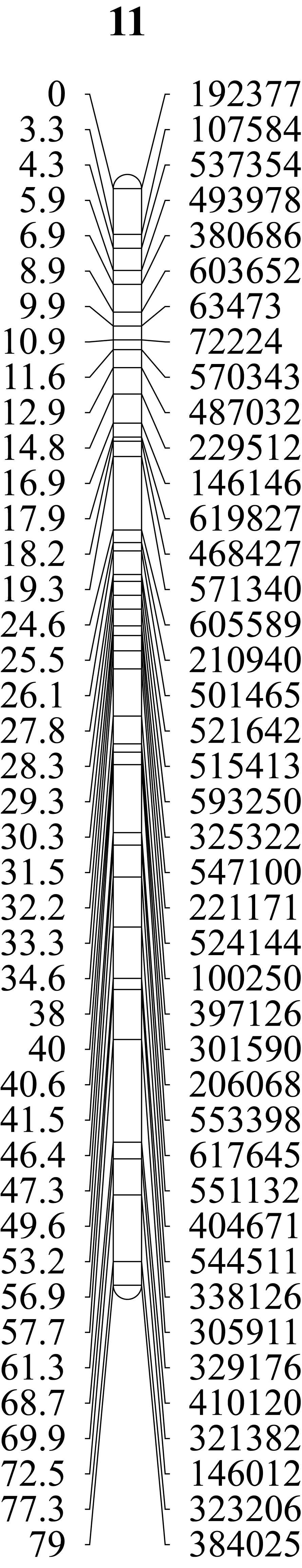

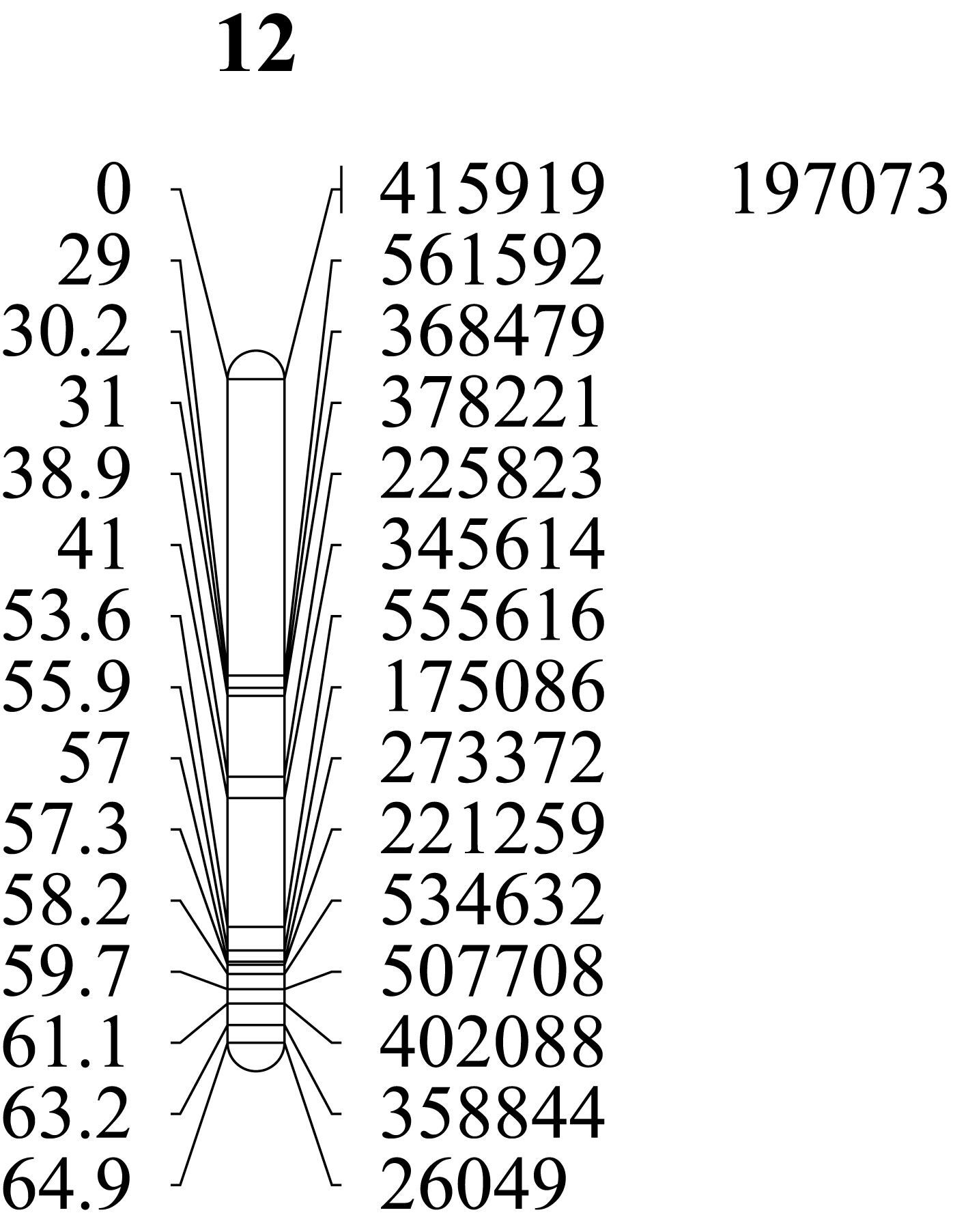

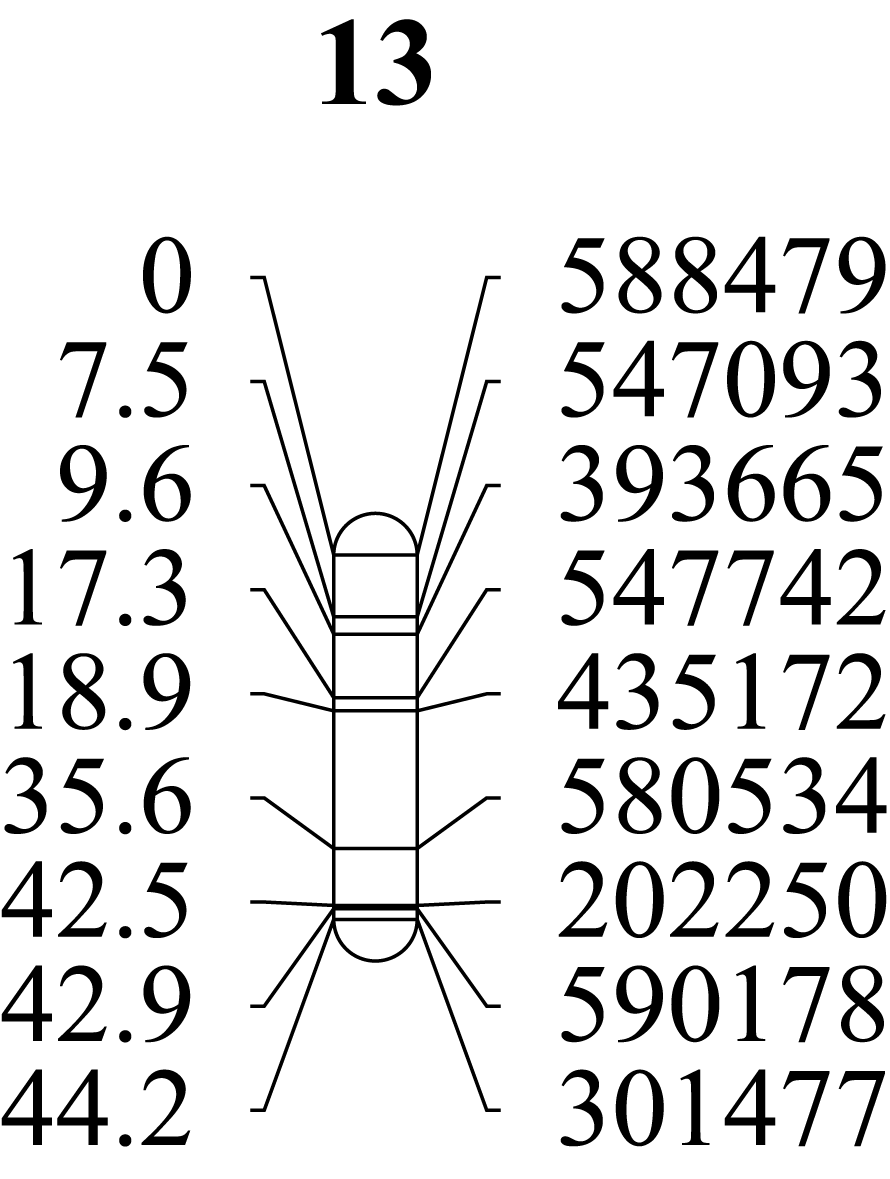

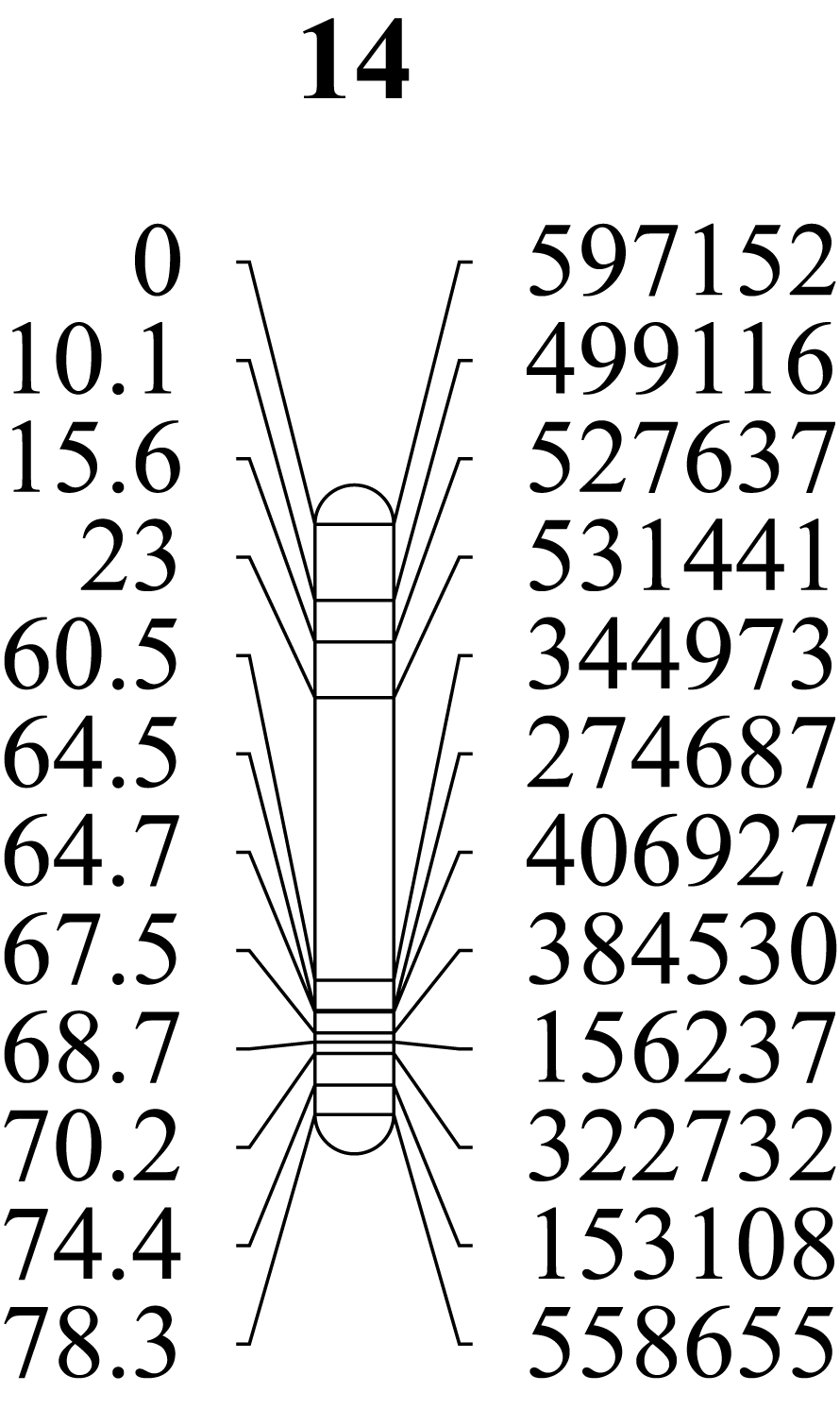

Supplement: Figure S1 — The female linkage map for Pinctada fucata . The map is composed of 14 linkage groups, with 558 markers, and spans 1024.3 cM. The map distances (in cM) are indicated on the left of the chromosomes and the names of the SNP markers are shown on the right. (DOC) [file pone.0111707.s001.doc]

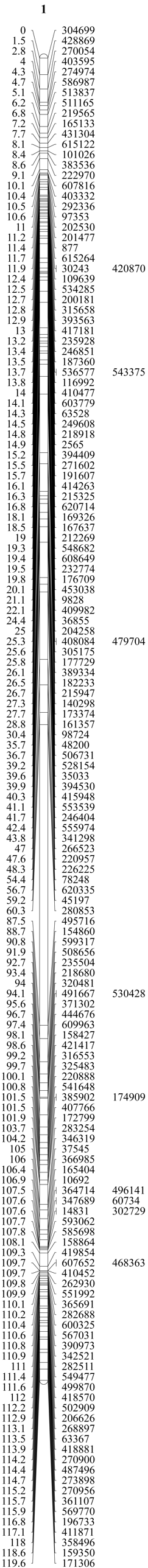

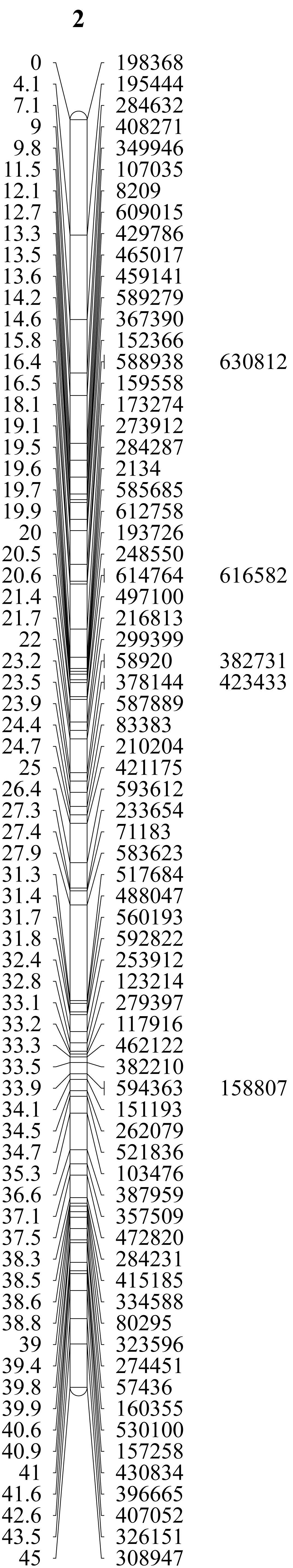

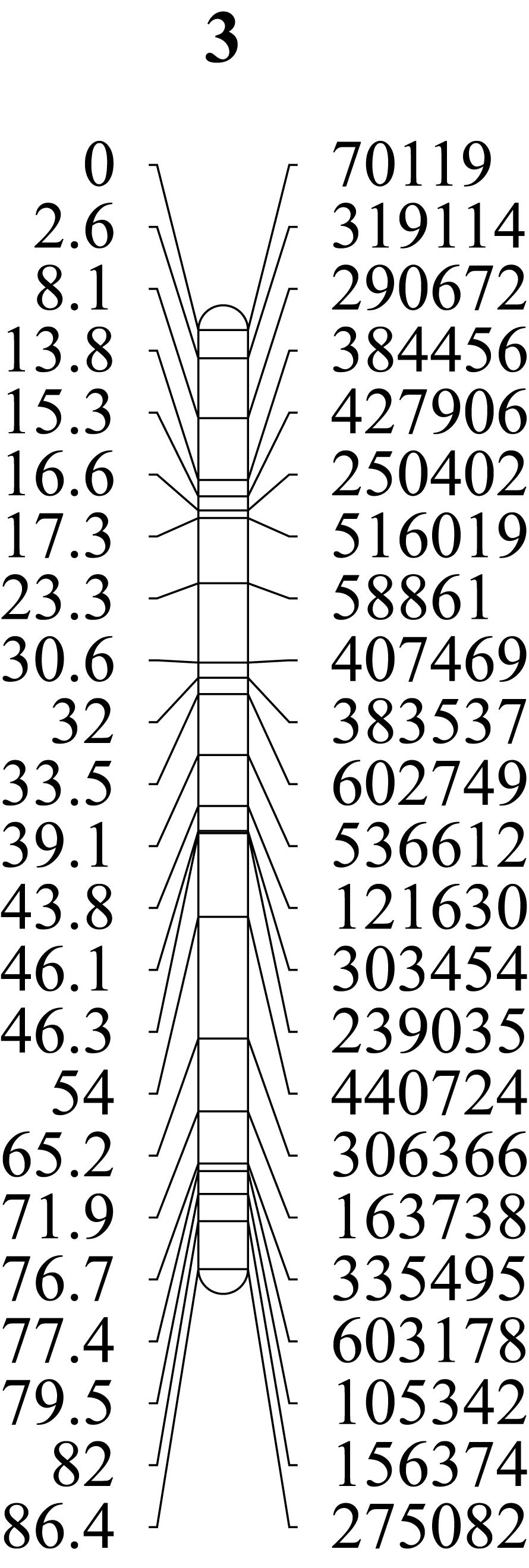

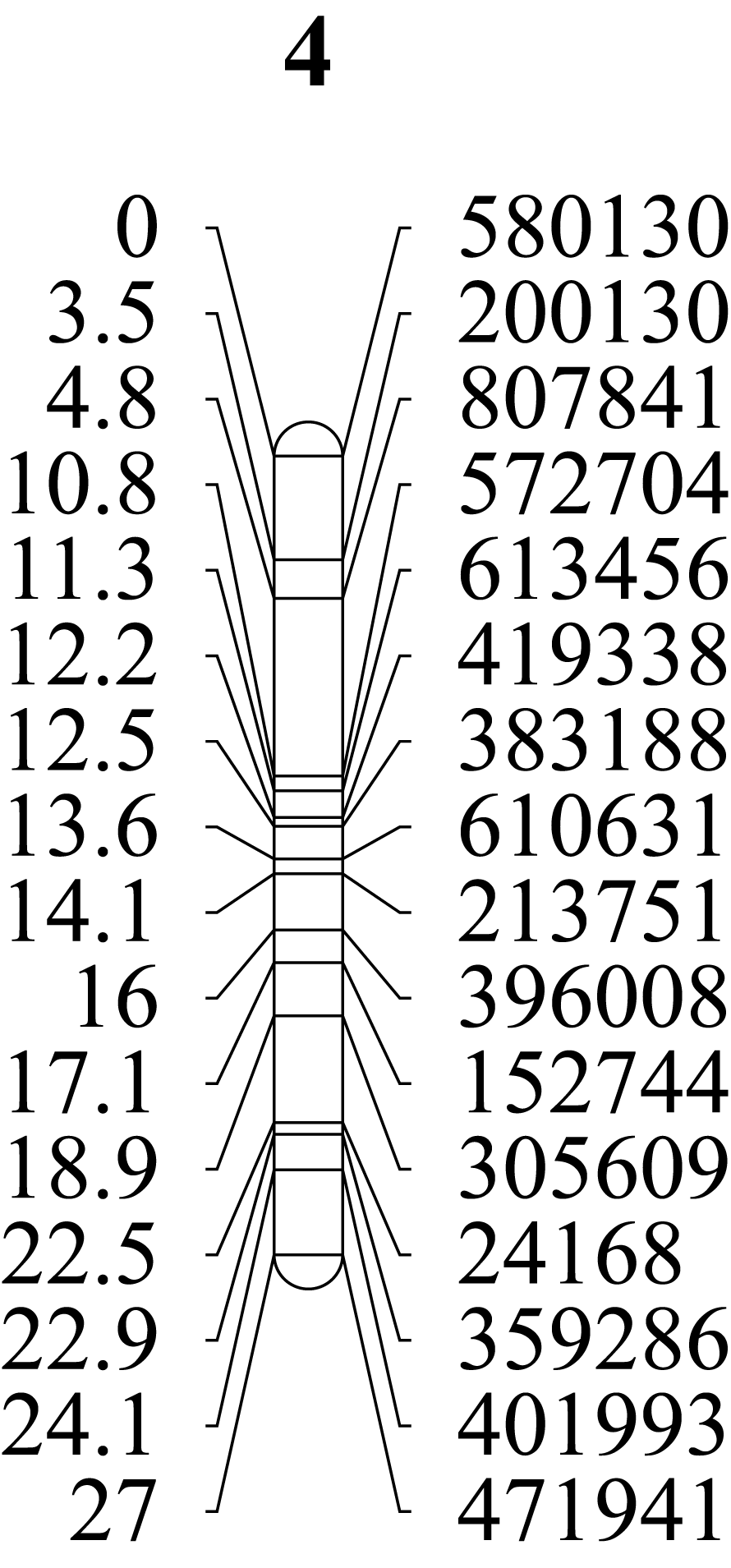


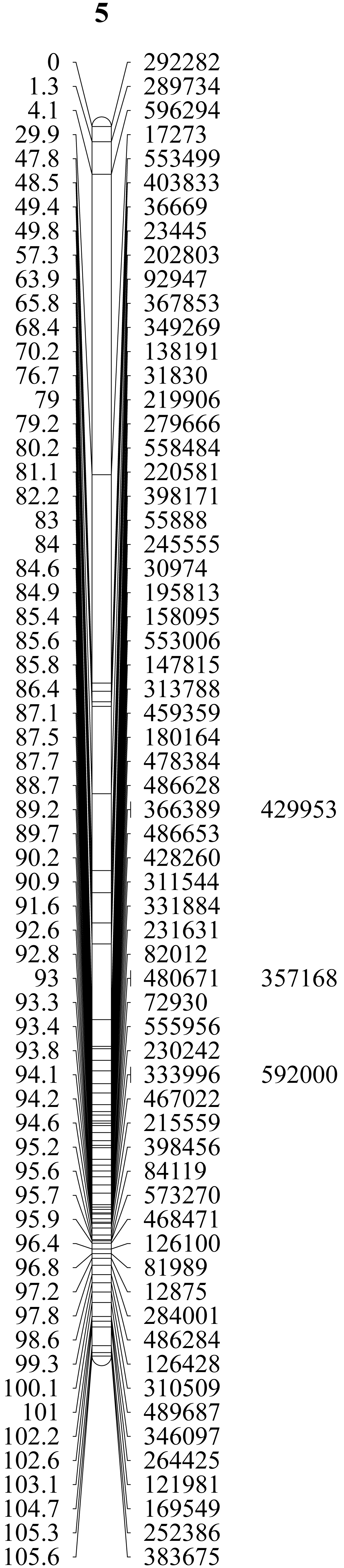

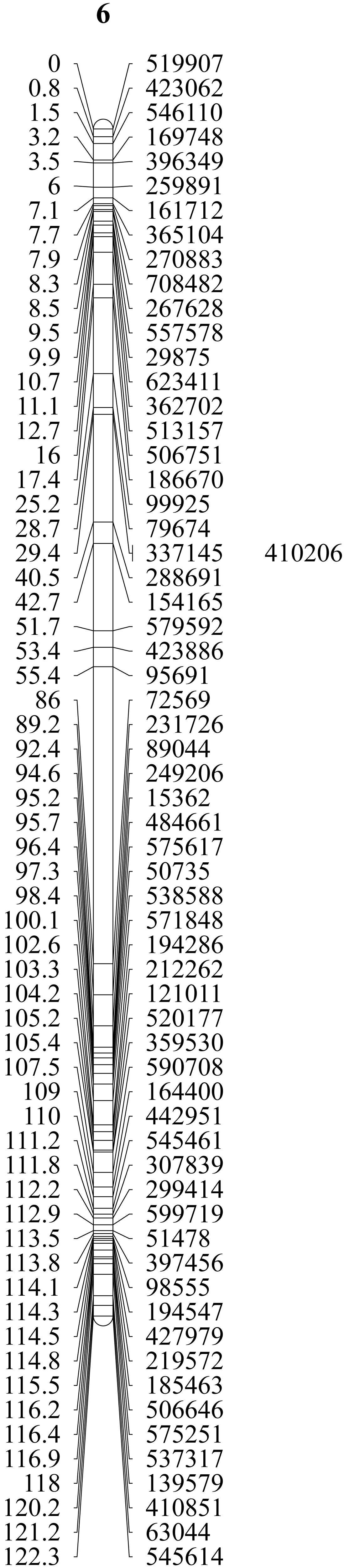

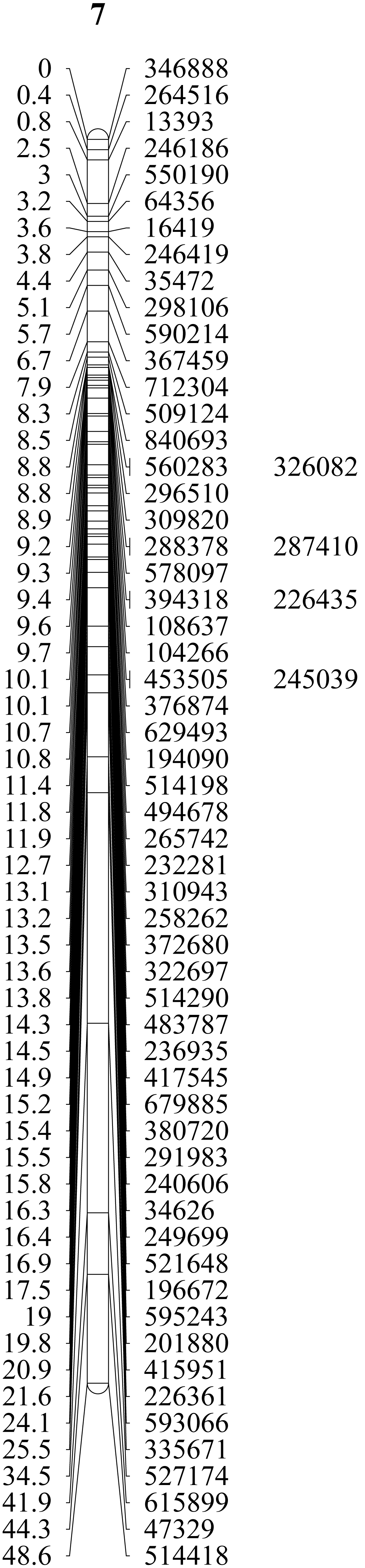

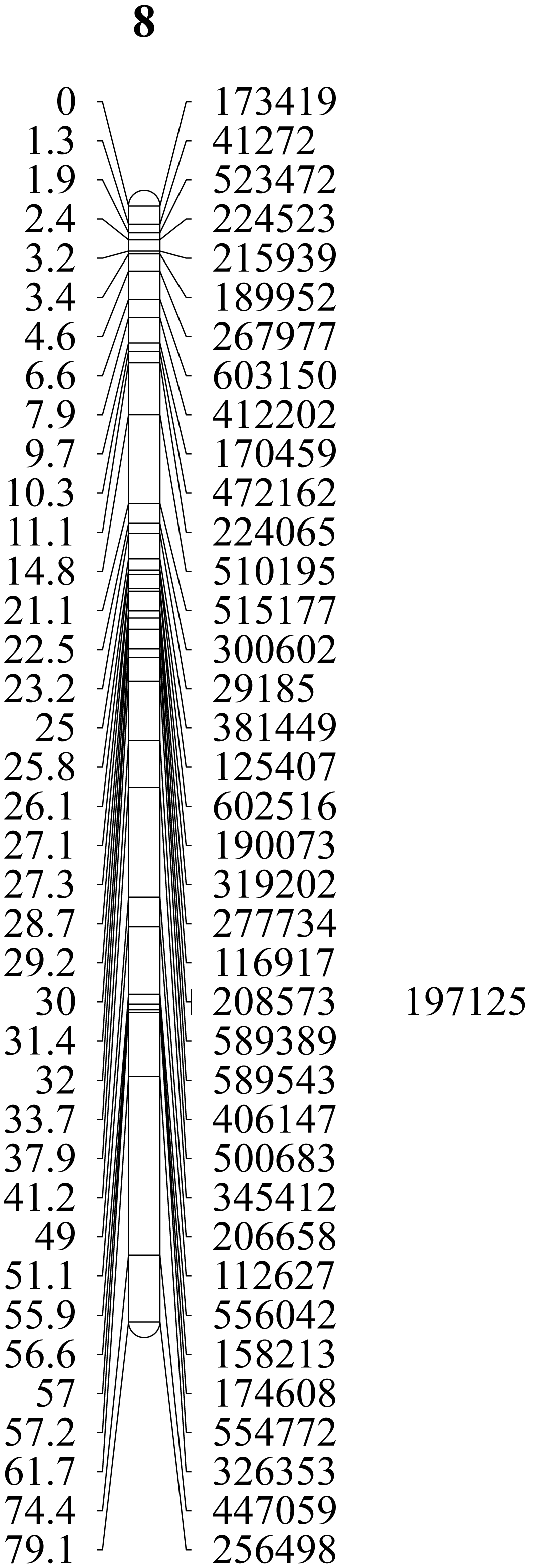


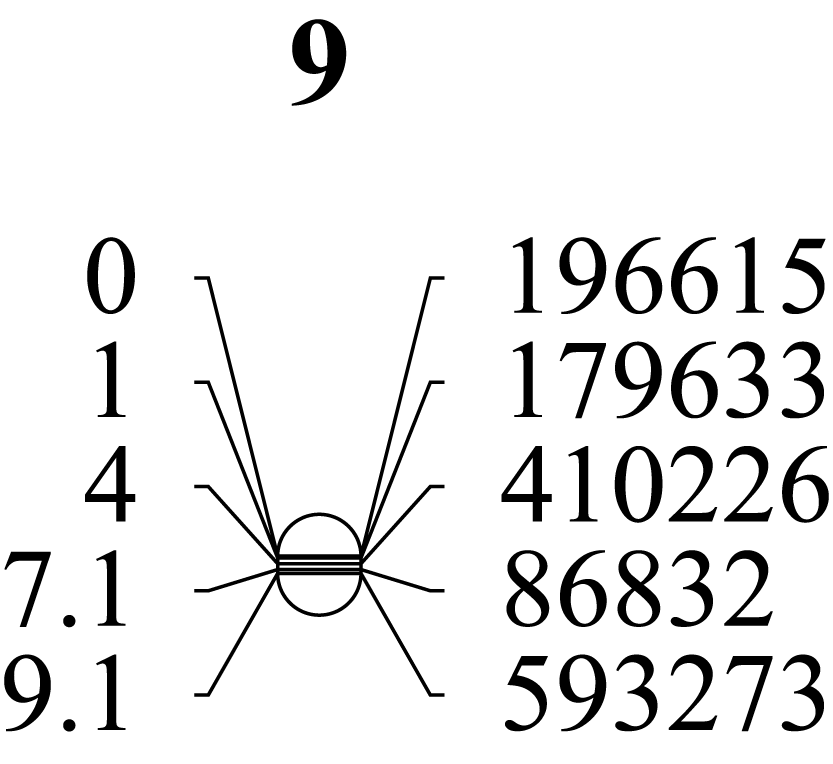

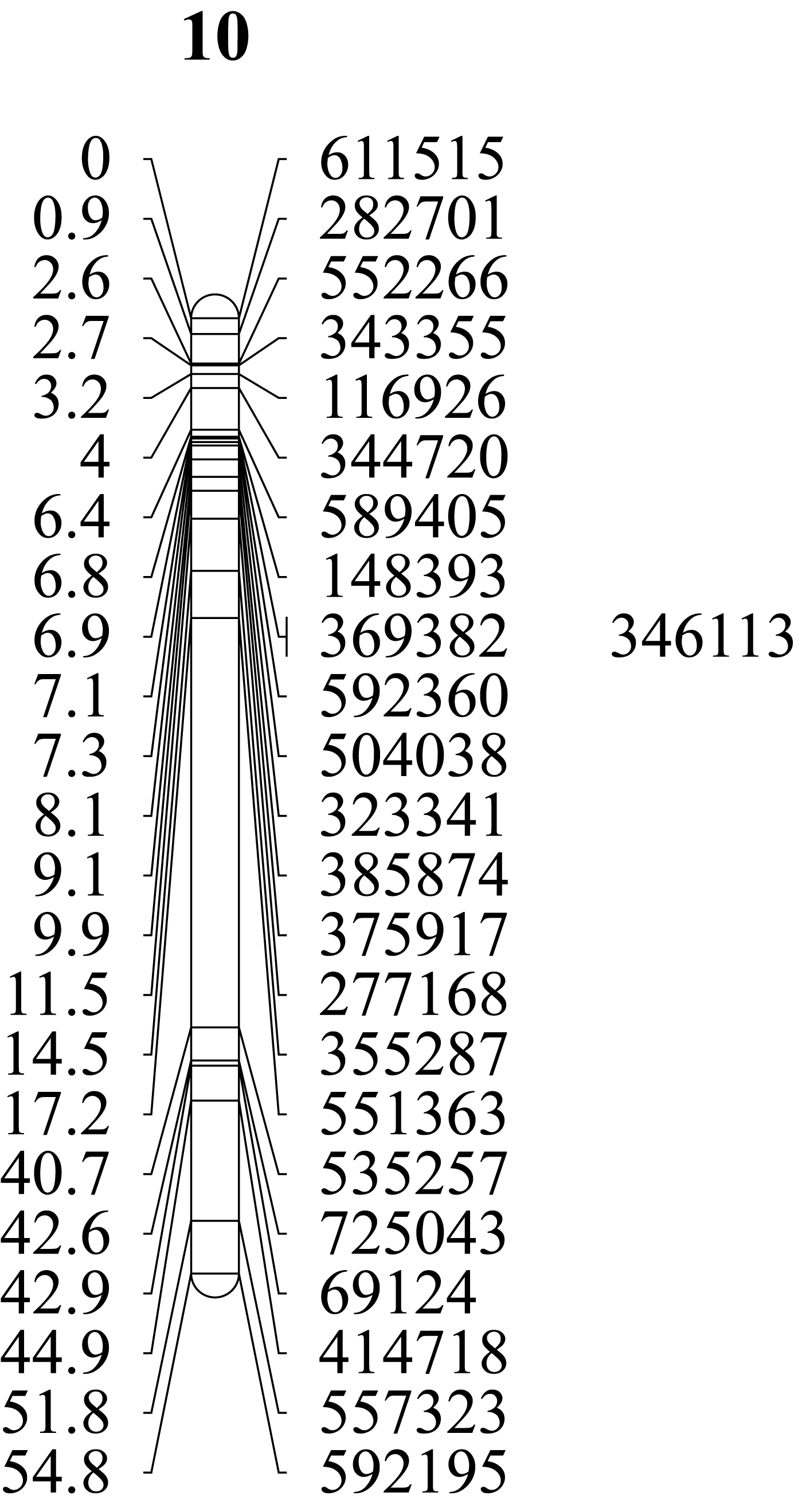

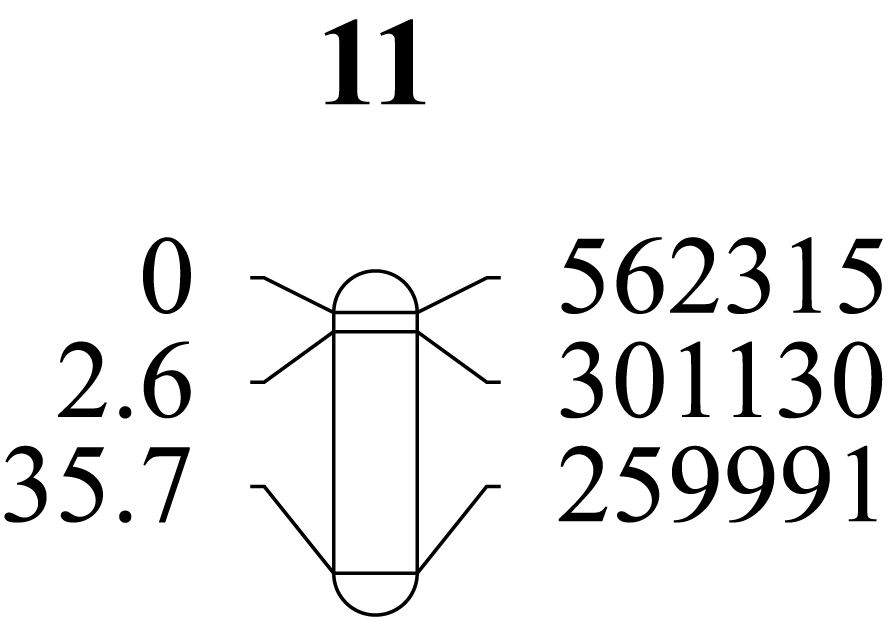

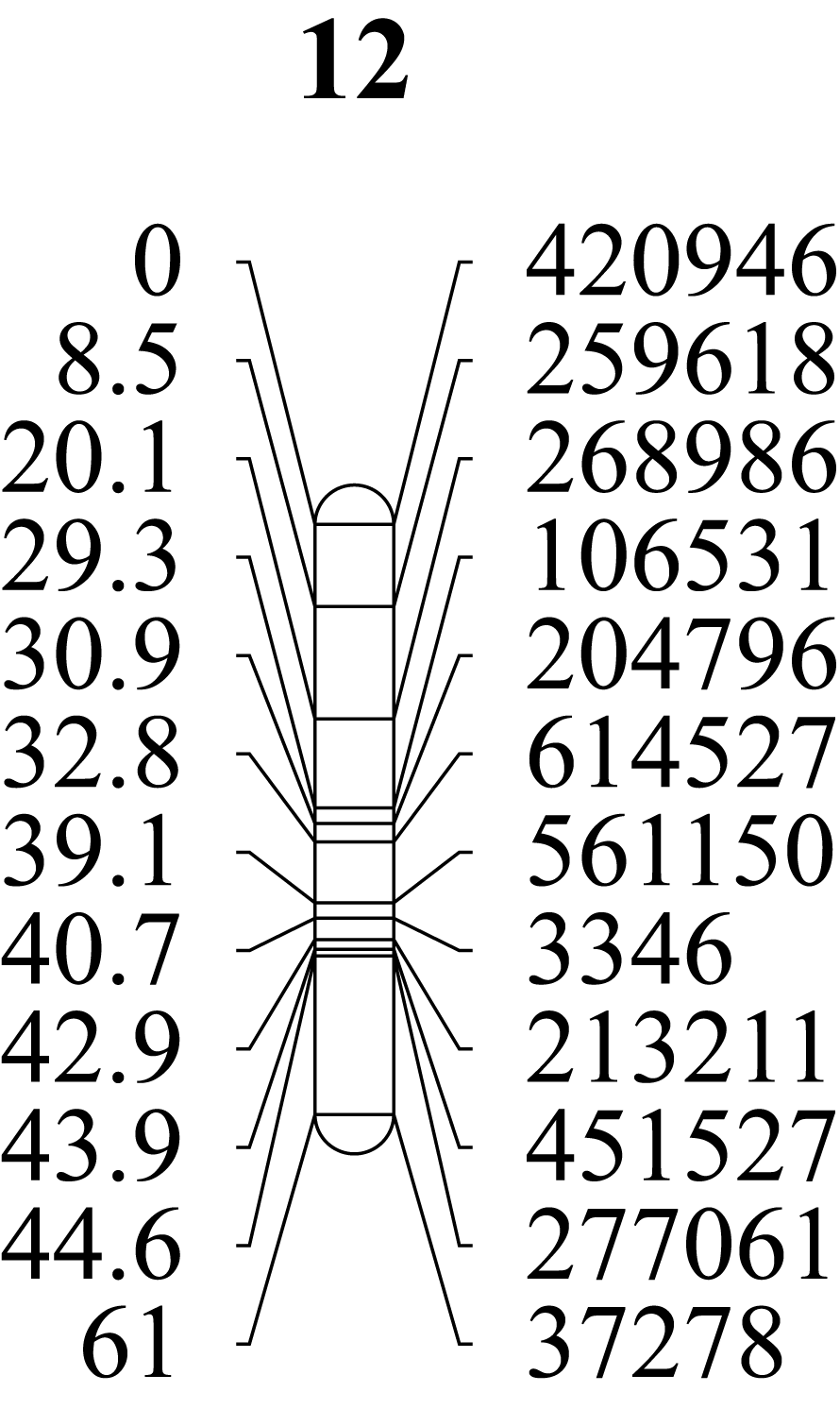

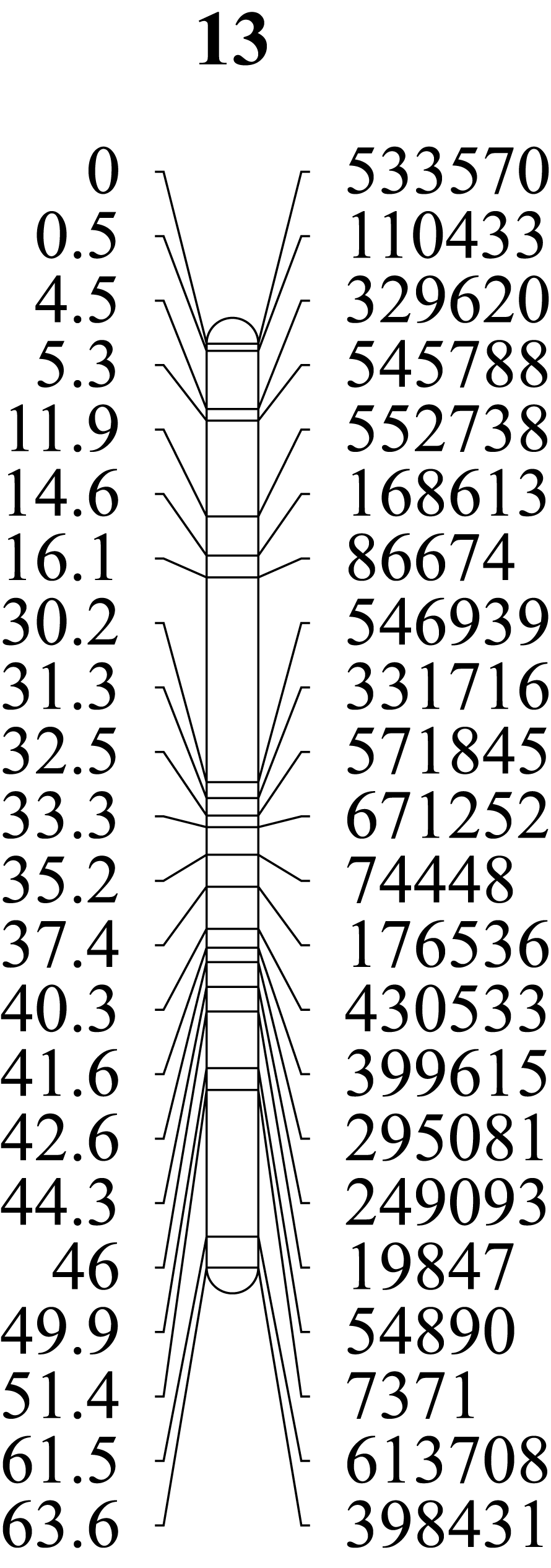

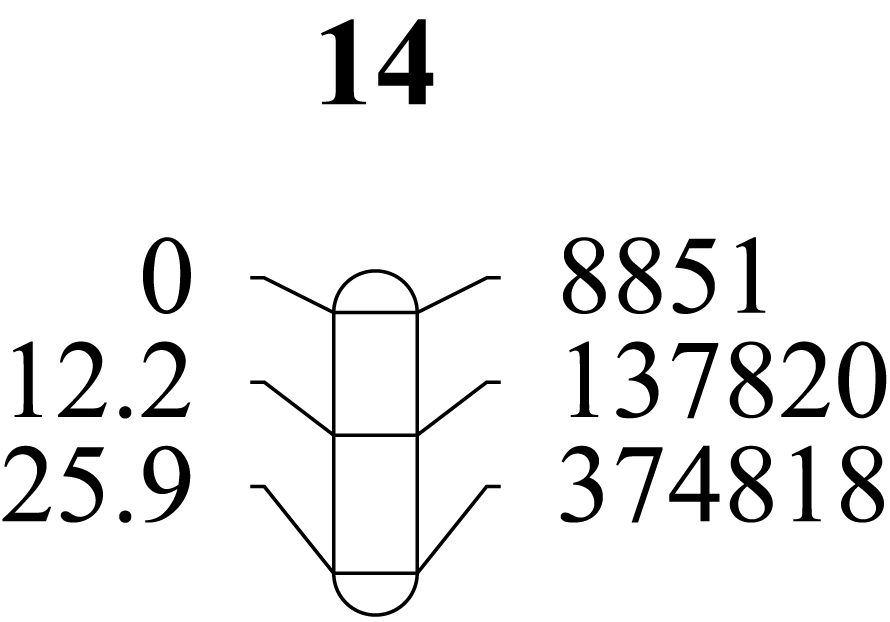

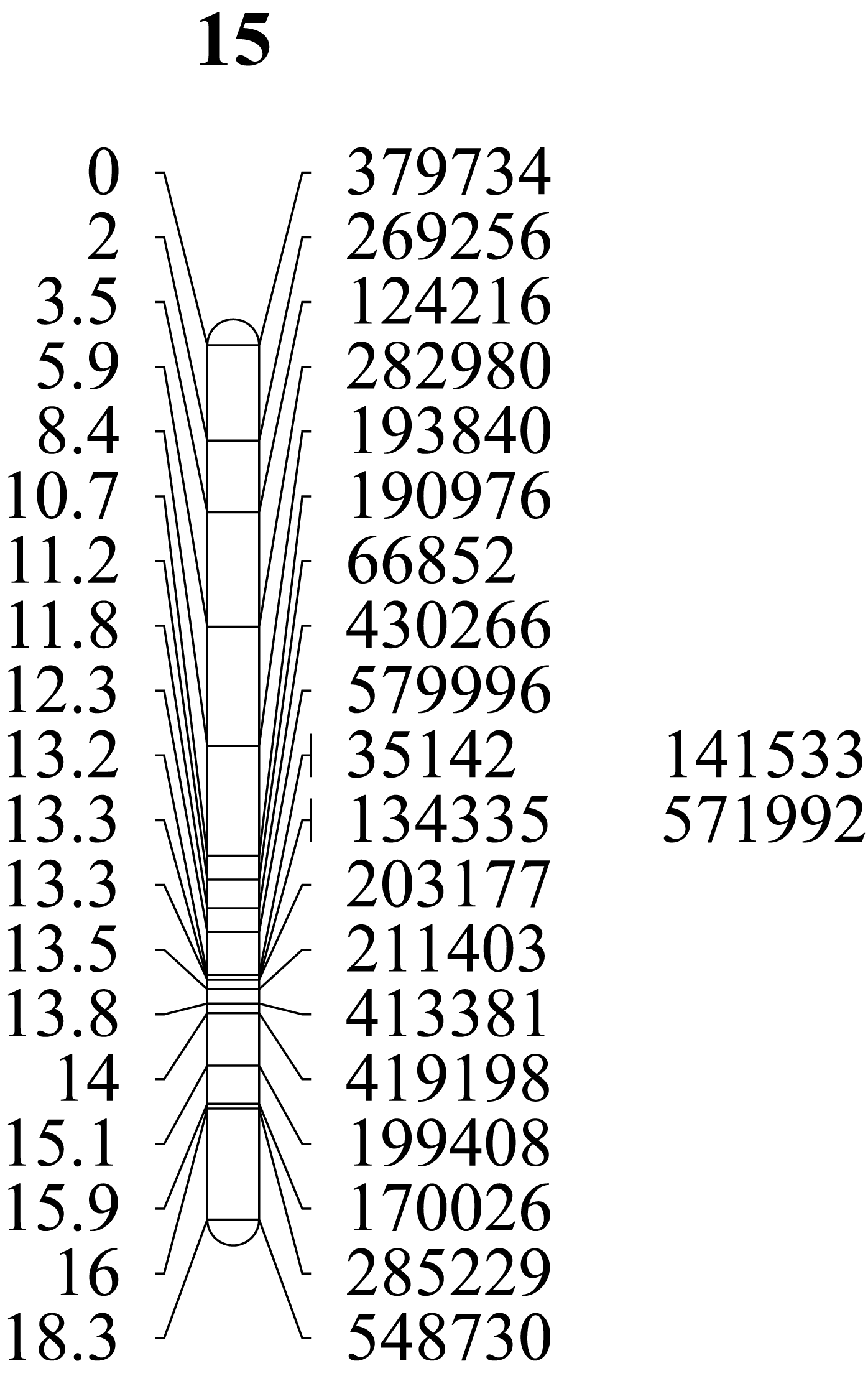

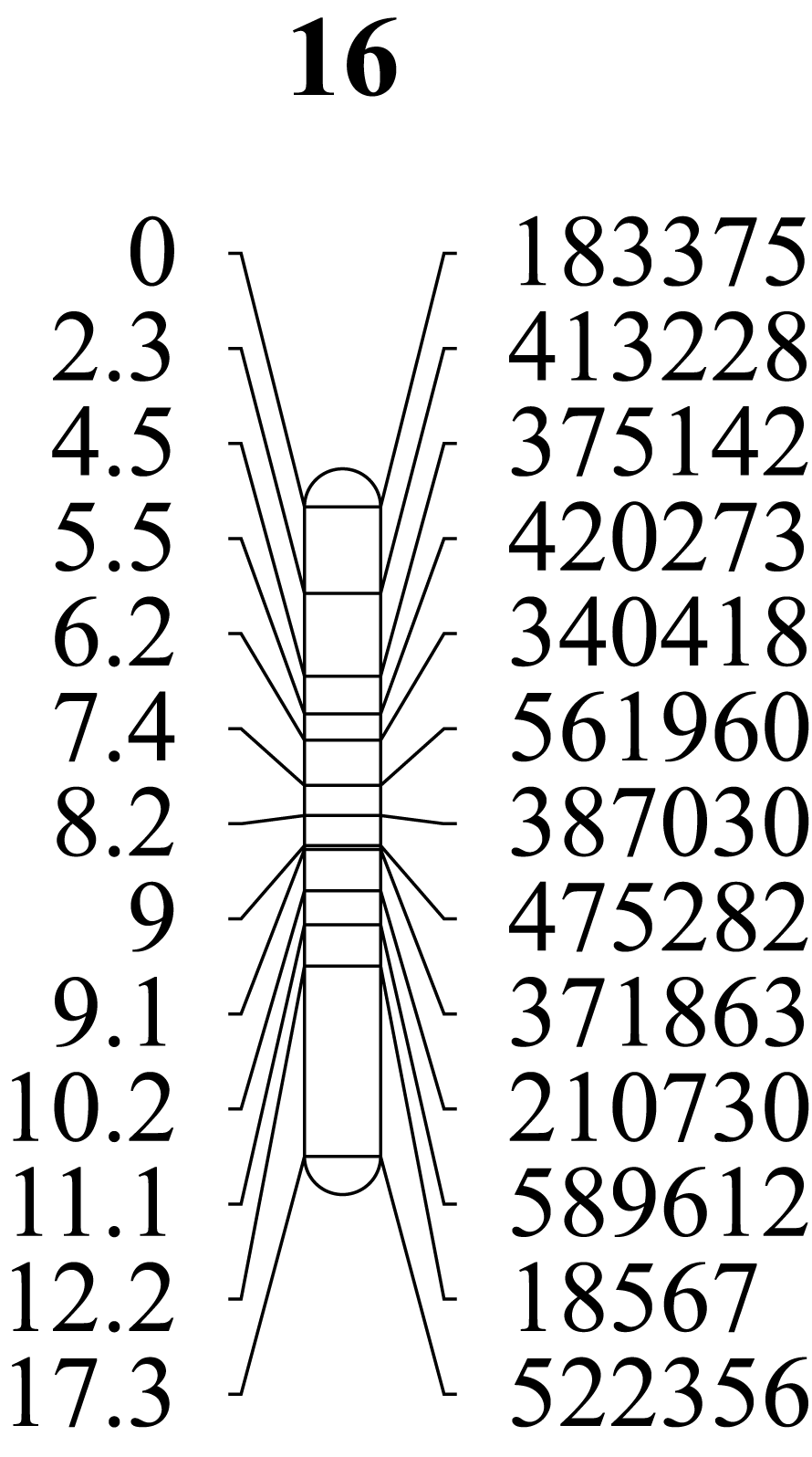

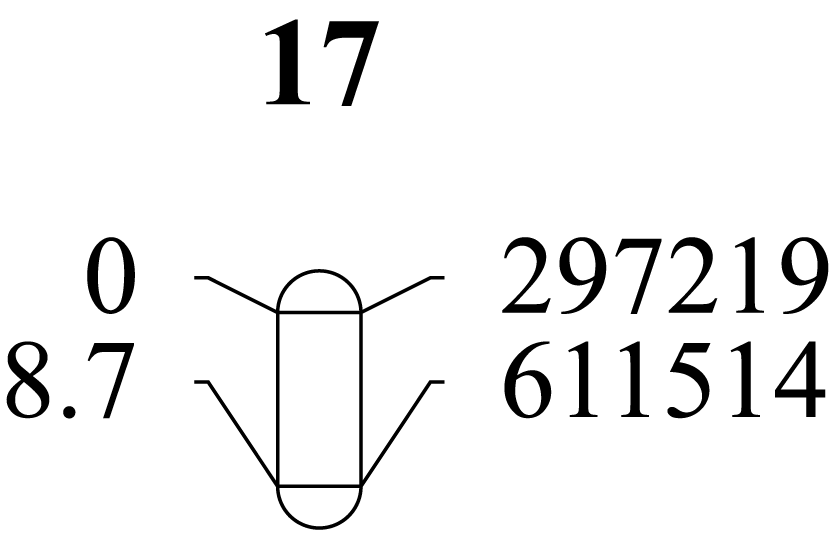

Supplement: Figure S2 — The male linkage map for Pinctada fucata . The map is composed of 17 linkage groups, with 607 markers, and spans 928.0 cM. The map distances (in cM) are indicated on the left of the chromosomes and the names of the SNP markers are shown on the right. (DOC) [file pone.0111707.s002.doc]
