# Supplementary material for: Genetic Mapping and QTL Analysis of Growth-Related Traits in Pinctada fucata Using Restriction-Site Associated DNA Sequencing
Source: PLoS One. 2014 Nov 4;9(11):e111707. doi: 10.1371/journal.pone.0111707 (PMC4219768; doi:10.1371/journal.pone.0111707)
Supplement: Table S1 — Statistics of sample information. Detailed statistics on 100 samples used as the mapping family. Q20-rate means the percentage of bases with a quality value ≥20 and Q30-rate means the percentage of bases with quality value ≥30. (DOC) [file pone.0111707.s003.doc]

**Table S1: Statistics of sample information.** Detailed statistics on 100 samples used as the mapping family. Q20-rate means the percentage of bases with a quality value ≥20 and Q30-rate means the percentage of bases with quality value ≥30.

| sample | Clean Reads(M) | Clean Bases(Gb) | GC_Rate(%) | Q20_Rate(%) | Q30_Rate(%) |
| --- | --- | --- | --- | --- | --- |
| Paternal | 25.43 | 1.09 | 33.05 | 99.03 | 97.57 |
| Maternal | 17.07 | 0.75 | 33.05 | 98.89 | 97.32 |
| 2 | 44.98 | 1.93 | 33.02 | 98.25 | 95.66 |
| 3 | 24.27 | 1.07 | 33.60 | 96.51 | 93.01 |
| 4 | 21.52 | 0.96 | 32.75 | 98.90 | 97.25 |
| 5 | 16.92 | 0.69 | 33.33 | 95.32 | 90.41 |
| 6 | 11.98 | 0.52 | 33.86 | 99.07 | 97.61 |
| 8 | 31.19 | 1.34 | 33.51 | 98.87 | 97.15 |
| 9 | 10.41 | 0.45 | 33.51 | 99.22 | 98.04 |
| 10 | 12.39 | 0.55 | 33.55 | 98.90 | 97.25 |
| 11 | 20.40 | 0.86 | 33.49 | 98.98 | 97.44 |
| 13 | 17.02 | 0.73 | 32.95 | 99.00 | 97.50 |
| 14 | 11.68 | 0.53 | 33.27 | 98.55 | 96.54 |
| 15 | 14.85 | 0.64 | 33.10 | 99.05 | 97.59 |
| 16 | 16.39 | 0.70 | 33.09 | 98.91 | 97.39 |
| 17 | 14.44 | 0.59 | 32.87 | 97.95 | 95.31 |
| 18 | 13.01 | 0.56 | 33.50 | 98.45 | 95.95 |
| 19 | 15.39 | 0.66 | 34.68 | 98.65 | 96.26 |
| 20 | 37.67 | 1.69 | 33.26 | 98.35 | 95.58 |
| 21 | 11.13 | 0.47 | 33.51 | 98.89 | 97.25 |
| 23 | 16.31 | 0.68 | 34.04 | 98.11 | 95.31 |
| 24 | 19.81 | 0.88 | 33.11 | 98.92 | 97.26 |
| 25 | 22.24 | 0.98 | 32.74 | 98.94 | 97.41 |
| 26 | 15.48 | 0.67 | 32.99 | 98.41 | 96.25 |
| 27 | 13.42 | 0.60 | 33.48 | 97.72 | 94.75 |
| 28 | 11.77 | 0.52 | 33.63 | 96.46 | 92.35 |
| 29 | 17.78 | 0.76 | 32.91 | 98.63 | 96.82 |
| 30 | 25.16 | 1.05 | 32.96 | 99.01 | 97.59 |
| 31 | 29.62 | 1.21 | 33.70 | 98.79 | 97.09 |
| 32 | 24.45 | 1.07 | 33.06 | 98.86 | 97.26 |
| 33 | 15.78 | 0.68 | 33.27 | 98.95 | 97.39 |
| 34 | 31.91 | 1.44 | 33.37 | 98.97 | 97.32 |
| 36 | 15.58 | 0.66 | 33.26 | 99.18 | 97.95 |
| 37 | 14.28 | 0.63 | 33.74 | 99.10 | 97.90 |
| 39 | 28.12 | 1.24 | 33.45 | 98.95 | 97.31 |
| 40 | 24.33 | 1.07 | 33.78 | 98.98 | 97.40 |
| 41 | 15.53 | 0.65 | 33.30 | 98.77 | 97.14 |
| 43 | 18.74 | 0.80 | 33.24 | 99.11 | 97.90 |
| 44 | 15.98 | 0.69 | 33.15 | 99.05 | 97.98 |
| 45 | 20.68 | 0.88 | 33.00 | 99.10 | 97.75 |
| 46 | 14.85 | 0.63 | 33.50 | 98.90 | 97.63 |
| 47 | 18.69 | 0.84 | 32.95 | 98.95 | 97.34 |
| 48 | 14.57 | 0.62 | 33.40 | 99.17 | 98.21 |
| 49 | 15.59 | 0.69 | 33.83 | 99.08 | 98.08 |
| 50 | 15.77 | 0.69 | 33.40 | 99.06 | 98.04 |
| 51 | 19.28 | 0.83 | 32.80 | 98.98 | 97.46 |
| 52 | 18.44 | 0.80 | 33.70 | 99.17 | 98.03 |
| 53 | 28.95 | 1.27 | 34.56 | 99.02 | 97.52 |
| 54 | 31.67 | 1.39 | 34.47 | 98.99 | 97.45 |
| 56 | 22.62 | 0.95 | 34.78 | 98.90 | 97.42 |
| 57 | 13.80 | 0.61 | 33.49 | 99.07 | 98.10 |
| 58 | 15.41 | 0.65 | 33.40 | 99.08 | 98.10 |
| 59 | 14.18 | 0.58 | 33.31 | 99.25 | 98.33 |
| 60 | 16.82 | 0.70 | 33.49 | 98.99 | 97.61 |
| 64 | 21.24 | 0.88 | 32.99 | 99.23 | 98.23 |
| 65 | 18.27 | 0.76 | 33.40 | 99.13 | 97.83 |
| 66 | 16.50 | 0.70 | 33.07 | 98.89 | 97.50 |
| 67 | 33.41 | 1.47 | 33.27 | 98.38 | 95.98 |
| 68 | 36.23 | 1.59 | 33.97 | 98.66 | 96.57 |
| 69 | 27.63 | 1.24 | 32.90 | 98.96 | 97.36 |
| 70 | 14.10 | 0.58 | 33.57 | 99.16 | 98.05 |
| 71 | 16.03 | 0.69 | 33.00 | 99.03 | 97.61 |
| 72 | 17.24 | 0.75 | 32.89 | 98.59 | 96.60 |
| 73 | 16.93 | 0.73 | 32.94 | 98.96 | 97.42 |
| 74 | 16.40 | 0.73 | 33.60 | 98.68 | 97.03 |
| 75 | 20.47 | 0.85 | 32.52 | 99.16 | 97.90 |
| 78 | 15.21 | 0.68 | 33.62 | 99.17 | 98.03 |
| 79 | 14.39 | 0.63 | 33.45 | 99.19 | 98.10 |
| 80 | 17.09 | 0.75 | 33.24 | 99.16 | 98.04 |
| 81 | 12.53 | 0.53 | 33.30 | 99.28 | 98.29 |
| 82 | 16.49 | 0.70 | 33.29 | 99.21 | 98.15 |
| 84 | 19.46 | 0.80 | 33.07 | 99.26 | 98.21 |
| 85 | 14.25 | 0.63 | 34.02 | 98.64 | 96.76 |
| 86 | 16.77 | 0.72 | 33.50 | 99.09 | 97.72 |
| 87 | 38.60 | 1.62 | 34.03 | 98.67 | 96.90 |
| 88 | 20.78 | 0.89 | 33.35 | 99.11 | 97.86 |
| 89 | 20.63 | 0.88 | 33.47 | 98.91 | 97.44 |
| 90 | 19.06 | 0.82 | 32.70 | 98.99 | 97.48 |
| 91 | 21.17 | 0.94 | 33.17 | 99.14 | 97.83 |
| 92 | 17.24 | 0.75 | 33.07 | 98.98 | 97.47 |
| 93 | 38.45 | 1.69 | 33.64 | 98.93 | 97.36 |
| 95 | 32.10 | 1.35 | 33.46 | 98.90 | 97.40 |
| 96 | 13.25 | 0.54 | 33.61 | 98.83 | 97.34 |
| 97 | 25.18 | 1.05 | 32.90 | 99.09 | 97.76 |
| 98 | 15.84 | 0.66 | 33.72 | 99.06 | 97.78 |
| 99 | 20.40 | 0.90 | 33.16 | 98.88 | 97.28 |
| 100 | 34.88 | 1.53 | 33.82 | 98.35 | 96.02 |
| 101 | 15.66 | 0.69 | 33.87 | 98.72 | 97.19 |
| 102 | 14.01 | 0.59 | 34.34 | 97.97 | 95.38 |
| 103 | 19.03 | 0.82 | 33.22 | 98.83 | 97.21 |
| 104 | 16.84 | 0.71 | 33.67 | 98.71 | 97.14 |
| 105 | 14.23 | 0.61 | 33.48 | 99.25 | 98.28 |
| 106 | 15.24 | 0.69 | 33.65 | 99.21 | 98.17 |
| 107 | 11.50 | 0.51 | 33.53 | 99.20 | 98.20 |
| 108 | 14.58 | 0.63 | 33.45 | 99.24 | 98.24 |
| 109 | 18.90 | 0.81 | 33.80 | 99.19 | 98.11 |
| 110 | 13.96 | 0.57 | 33.51 | 99.30 | 98.46 |
| 111 | 12.83 | 0.53 | 33.12 | 99.35 | 98.49 |
| 112 | 23.23 | 1.00 | 33.84 | 99.05 | 97.79 |
| 114 | 22.55 | 1.01 | 33.82 | 99.00 | 97.64 |
